# Regulatory T Cells in Health and Disease

**DOI:** 10.1002/mco2.71107

**Published:** 2026-11-09

**Authors:** Mingyi Li, Si Zhang, Tangchun Wu, Tingting Tang, Xiang Cheng

**Affiliations:** ^1^ Department of Cardiology Union Hospital, Tongji Medical College, Huazhong University of Science and Technology Wuhan China; ^2^ Hubei Key Laboratory of Biological Targeted Therapy Union Hospital, Tongji Medical College, Huazhong University of Science and Technology Wuhan China; ^3^ Hubei Provincial Engineering Research Center of Immunological Diagnosis and Therapy for Cardiovascular Diseases Union Hospital, Tongji Medical College, Huazhong University of Science and Technology Wuhan China; ^4^ Department of Occupational and Environmental Health School of Public Health Tongji Medical College, Huazhong University of Science and Technology Wuhan China

**Keywords:** cell plasticity, cell‐based therapy, immune homeostasis, regulatory T cells, tissue adaptation

## Abstract

Regulatory T cells (Tregs) are central guardians of immune tolerance and tissue homeostasis. Initially defined as suppressors of excessive immune responses, Tregs are now recognized as adaptable cells that integrate antigenic, cytokine, metabolic, and tissue‐derived signals to coordinate immune regulation, inflammation resolution, and tissue repair. However, these dimensions are often considered separately, and their interrelationships across physiological and pathological contexts remain insufficiently integrated. Here, we summarize the developmental origin, lineage stability, plasticity, and tissue adaptation of Tregs, and examine how these features shape their context‐dependent functions. In lymphoid organs, Tregs maintain systemic tolerance by limiting antigen‐presenting cell activation, restraining IL‐2 availability, and setting the threshold for T‐ and B‐cell responses, whereas in non‐lymphoid tissues, they acquire organ‐specific transcriptional, metabolic, and migratory programs that support barrier integrity, metabolic fitness, and regenerative repair. Chronic inflammation, persistent antigenic stimulation, oxidative stress, and metabolic disturbance can in turn disrupt these programs, promoting functional reprogramming or lineage instability, while excessive Treg‐mediated suppression may favor pathogen persistence and tumor immune escape. We further discuss Treg alterations across major disease settings and emerging Treg‐targeted therapies, providing an integrated framework that links developmental origin, tissue context, and functional state to more precise therapeutic strategies.

## Graphical abstract

Regulatory T cells (Tregs) arise through distinct developmental pathways and adapt to specialized tissue niches to maintain immune tolerance, barrier integrity, and metabolic homeostasis. In disease, Tregs undergo context‐dependent changes, ranging from functional insufficiency and lineage instability in autoimmune, inflammatory, cardiovascular, and transplant disorders to enhanced immunosuppression and metabolic adaptation in tumors. Understanding these divergent Treg states provides a framework for precision therapies that either restore or augment protective Tregs or selectively eliminate and reprogram tumor‐associated Tregs (figure was created with Biorender.com).

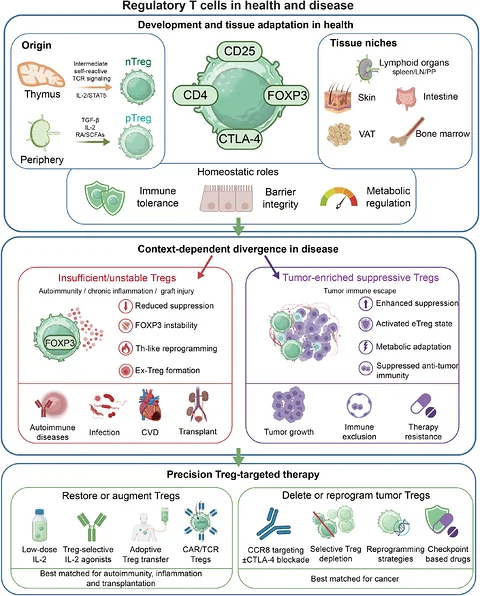

## 1 Introduction

Regulatory T cells (Tregs) were first identified by Sakaguchi and colleagues in 1995 as a specialized subset of CD4^+^ T lymphocytes with immunosuppressive activity [1]. They are now commonly characterized by expression of the transcription factor forkhead box P3 (FOXP3) [2]. Tregs constitute approximately 5%–10% of the total CD4^+^ T‐cell compartment and typically express CD25 and cytotoxic T‐lymphocyte‐associated antigen 4 (CTLA‐4), together with low or absent levels of CD127 [3]. Functionally, they are indispensable for maintaining immune tolerance, preventing autoimmunity, limiting inflammation, and facilitating tissue repair [4]. Beyond the canonical CD4^+^FOXP3^+^ lineage, additional Treg populations have been described, including interleukin‐10 (IL‐10)^+^FOXP3^−^ type 1 regulatory T cells (Tr1 cells), transforming growth factor‐β (TGF‐β)^+^FOXP3^−^ T helper type 3 (Th3) cells, CD8^+^ suppressor T cells, and certain natural killer regulatory populations. Some CD4^−^CD8^−^ T cells and γδ T cells have also been reported to exert immunoregulatory functions in specific contexts [5]. Because CD4^+^FOXP3^+^ Tregs are the most extensively studied population, this review focuses primarily on this canonical lineage, referred to hereafter as Tregs.

Traditionally, Tregs have been viewed as immunosuppressive cells that restrain excessive activation of effector T cells and antigen‐presenting cells (APCs) by secreting IL‐10 and TGF‐β, modulating costimulation through CTLA‐4, competing for IL‐2, and engaging other suppressive pathways [3]. In lymphoid organs, Tregs maintain systemic immune tolerance and set immune‐activation thresholds, enabling the host to avoid autoreactivity and excessive inflammation while preserving effective immune defense [3, 4]. Accumulating evidence indicates that, beyond these classical immunosuppressive functions, Tregs can acquire tissue‐specific transcriptional and migratory programs in non‐lymphoid tissues, allowing them to adapt to local microenvironments and contribute to organ homeostasis [6, 7]. Treg function is therefore highly context dependent. During acute stress or tissue injury, these homeostatic programs can be mobilized to promote inflammation resolution and tissue repair [8, 9, 10, 11]. By contrast, chronic inflammation, oxidative stress, or metabolic disturbance may induce phenotypic instability and functional reprogramming [12]. Some Tregs with reduced FOXP3 expression may adopt interferon‐γ (IFN‐γ)^+^ T helper 1 (Th1)‐like or IL‐17^+^ Th17‐like pathogenic states or lose FOXP3 expression entirely, forming ex‐Tregs that promote local inflammation and tissue injury [13, 14]. This plasticity highlights both the complexity of Treg regulation under pathological conditions and the challenges it poses for therapeutic applications.

In recent years, several Treg‐based therapeutic strategies, such as in vivo Treg expansion or adoptive Treg transfer, have shown promise in autoimmune diseases and transplantation and are increasingly being explored in chronic inflammatory diseases [15, 16, 17]. A comprehensive understanding of Treg tissue adaptation, functional states, and dynamic fate transitions in health and disease is therefore essential for developing precision immunomodulatory strategies.

Accordingly, this review follows the biological trajectory of Tregs from their developmental origin to tissue adaptation, pathological remodeling and therapeutic manipulation. We first discuss thymic and peripheral Treg development, lineage stability and the relative contributions of thymus‐derived and peripherally induced Treg populations. We then examine how Tregs traffic to and adapt within lymphoid and non‐lymphoid tissues under physiological conditions, emphasizing the anatomical and molecular mechanisms that support immune tolerance and tissue homeostasis. Next, we consider how these regulatory programs are altered in disease, focusing on autoimmune and immune‐mediated inflammatory disorders, transplantation, cardiovascular diseases (CVDs), infectious diseases, and cancer, and highlighting the consequences of altered recruitment, functional adaptation and lineage instability. Finally, we summarize representative clinical trials of Treg‐directed interventions and discuss the translational principles that are emerging from these studies. By linking developmental origin, tissue context and disease‐associated functional state with therapeutic strategy, this review aims to provide an integrated framework for understanding Treg biology in health and disease and for guiding the development of more precise Treg‐targeted therapies.

## 2 Developmental Origins of Tregs

Treg developmental origin provides the foundation for their antigen recognition and functional stability. Thymic and peripheral pathways generate Treg populations with distinct developmental features, while differences in their contribution across tissues and developmental stages further shape Treg heterogeneity.

### 2.1 Ontogeny of Tregs

Tregs constitute a functionally mature and distinct lineage of T lymphocytes [3] and arise through a developmental continuum extending from the thymus to the periphery (Figure 1). Based on their site of origin, Tregs are broadly classified as thymus‐derived natural Tregs (nTregs) or peripherally induced Tregs (pTregs); pTregs can also be generated in vitro (Table 1). In mice, Hsieh et al. proposed a two‐step model of nTreg development. In the first step, CD4^+^ thymocytes bearing TCRs with intermediate affinity for self‐peptide‐major histocompatibility complex (MHC) class II complexes differentiate into CD25^+^FOXP3^−^ nTreg precursors in response to TCR stimulation, accompanied by chromatin remodeling at the *Foxp3* locus. In the second step, these precursors become relatively independent of TCR signaling and upregulate FOXP3 in response to cytokines, primarily IL‐2 and, to a lesser extent, IL‐15. These signals induce demethylation and transcriptional activation of the *Foxp3* locus, generating mature CD25^+^FOXP3^+^ nTregs [18]. A parallel population of CD25^−^FOXP3^low^ thymic nTreg precursors has also been identified [19]. Owen et al. subsequently proposed a dual‐precursor model in which mature nTregs arise through two complementary pathways. The first is an IL‐2‐dependent CD25^+^FOXP3^−^ precursor pathway initiated by relatively strong self‐reactive TCR signaling that relies on IL‐2/STAT5 signaling for rapid differentiation into CD25^+^FOXP3^+^ nTregs with high self‐antigen affinity, lineage stability, and potent suppressive activity. The second begins with CD25^−^FOXP3^low^ precursors generated under relatively weak TCR stimulation and limited IL‐2 availability. Subsequent stimulation with IL‐2 or IL‐4 upregulates CD25 and further increases FOXP3 expression, ultimately producing mature nTregs. nTregs arising through the latter pathway exhibit broader antigen recognition, greater tissue adaptability, and greater phenotypic plasticity in peripheral tissues [20] (Figure 1A).

**FIGURE 1:**
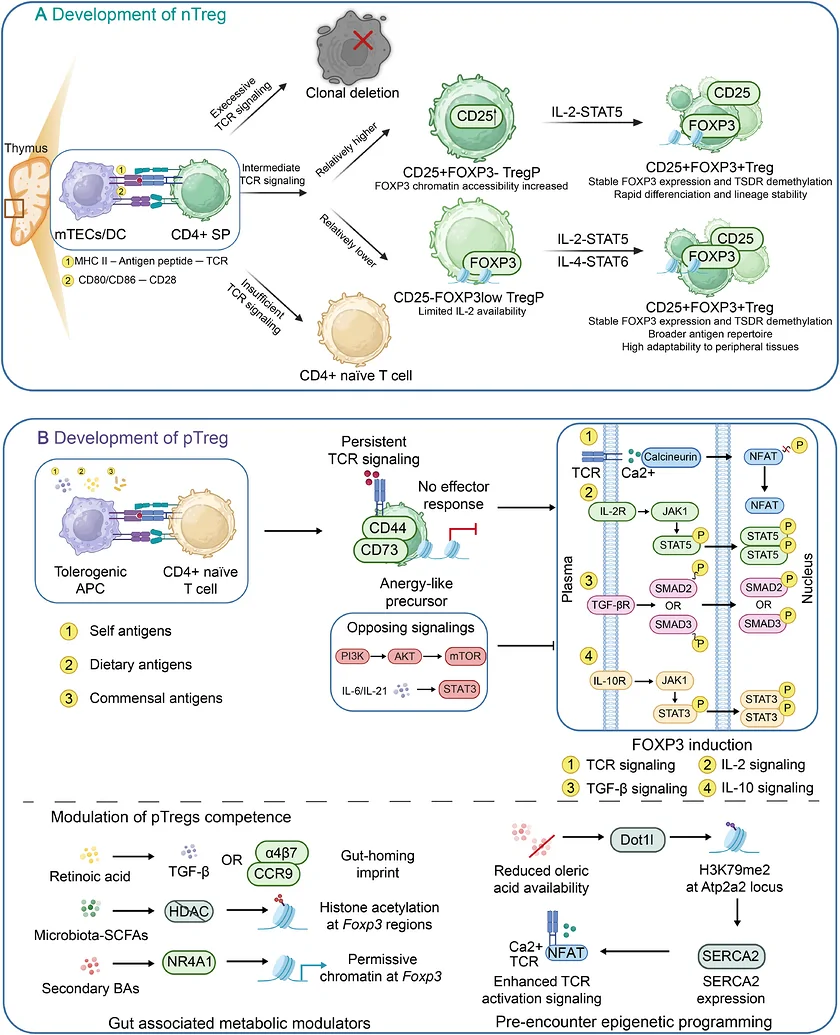
Development of thymic and peripheral regulatory T cells. (A) Thymic Treg development. Intermediate self‐reactive TCR signaling directs CD4^+^ single‐positive thymocytes toward the nTreg lineage, whereas excessive signaling induces clonal deletion. Relatively stronger TCR signaling generates CD25^+^FOXP3^−^ precursors that rapidly mature through IL‐2/IL‐15–STAT5 signaling, whereas relatively weaker signaling under limited IL‐2 availability generates CD25^−^FOXP3^low^ precursors that mature through IL‐2–STAT5 or IL‐4–STAT6 signaling. Both pathways generate lineage‐stable CD25^+^FOXP3^+^ nTregs with distinct antigen‐recognition and tissue‐adaptive properties. (B) Peripheral Treg development. Under tolerogenic conditions, recognition of self, dietary, or commensal antigens can generate anergy‐like CD4^+^ T‐cell precursors that subsequently acquire FOXP3. TCR–NFAT, IL‐2–STAT5, TGF‐β–SMAD2/3, and IL‐10–STAT3 signaling promote pTreg differentiation, whereas PI3K–AKT–mTOR and IL‐6/IL‐21–STAT3 signaling oppose this process. Retinoic acid, microbiota‐derived SCFAs, and secondary bile acids further enhance pTreg competence and gut adaptation. Reduced thymic oleic acid availability also enhances DOT1L–H3K79me2–SERCA2–Ca^2^^+^–NFAT signaling, preprogramming subsequent peripheral FOXP3 induction (figure was created with Biorender.com). AKT, protein kinase B; APC, antigen‐presenting cell; BAs, bile acids; CCR9, C‐C motif chemokine receptor 9; CD, cluster of differentiation; DC, dendritic cell; DOT1L, disruptor of telomeric silencing 1‐like; FOXP3, forkhead box P3; H3K79me2, histone H3 lysine 79 dimethylation; HDAC, histone deacetylase; IL, interleukin; IL‐2R, interleukin‐2 receptor; IL‐10R, interleukin‐10 receptor; JAK1, Janus kinase 1; mTEC, medullary thymic epithelial cell; MHC II, major histocompatibility complex class II; mTOR, mechanistic target of rapamycin; NFAT, nuclear factor of activated T cells; NR4A1, nuclear receptor subfamily 4 group A member 1; nTreg, natural regulatory T cell; PI3K, phosphatidylinositol 3‐kinase; pTreg, peripheral regulatory T cell; SCFA, short‐chain fatty acid; SERCA2, sarcoplasmic/endoplasmic reticulum calcium ATPase 2; SMAD, SMAD family member; SP, single‐positive; STAT, signal transducer and activator of transcription; TCR, T‐cell receptor; TGF‐β, transforming growth factor‐β; TGF‐βR, transforming growth factor‐β receptor; Treg, regulatory T cell; TregP, regulatory T‐cell precursor; TSDR, Treg‐specific demethylated region; α4β7, integrin alpha 4 beta 7.

**TABLE 1:** Comparison between nTregs and pTregs.

	nTregs	pTregs	Reference
Site of origin	Thymus	Peripheral	[18, 20, 24, 25]
TCR dependence	Intermediate signaling	Weak signaling	[18, 20, 24, 25]
Induction signals	IL‐2, IL‐4, and IL‐15	TGF‐β, IL‐2, RA, IL‐33, IL‐10, SCFAs, and bile acids	[18, 20, 26, 27, 28, 29, 30, 31, 41, 42, 43, 44, 45]
Antigen specificity	Self‐antigens	Self or environmental antigens such as food or commensal antigens	[18, 20, 21, 22, 23]
FOXP3 expression	High and stable	Low and unstable	[20, 56, 60, 61]
*Foxp3* TSDR demethylation	Complete	Partial	[56, 62]
Aging‐related changes	Decline with thymic involution	Increase with environmental antigen exposure	[22, 48, 49]

Abbreviations: FOXP3, forkhead box protein P3; IL, interleukin; nTreg, natural regulatory T cell; pTreg, peripheral regulatory T cell; RA, retinoic acid; SCFAs, short‐chain fatty acids; TCR, T cell receptor; TGF‐β, transforming growth factor‐β; TSDR, Treg‐specific demethylated region.

In peripheral tolerogenic environments, naïve CD4^+^FOXP3^−^ T cells differentiate into pTregs after recognizing self, dietary, or commensal antigens presented in the absence of strong inflammatory costimulation [21, 22, 23]. Antigen recognition may first establish an anergy‐like precursor state, from which selected antigen‐specific cells subsequently acquire FOXP3 expression [23]. pTreg differentiation depends on the integrated strength and duration of TCR signaling, costimulatory inputs, and the local cytokine milieu [24, 25]. TCR‐induced calcium–calcineurin–nuclear factor of activated T cells (NFAT) signaling cooperates with TGF‐β signaling mediated by SMAD family member 3 (SMAD3) at conserved noncoding sequence 1 (CNS1) of the *Foxp3* locus to initiate transcription [26, 27]. IL‐2 signaling through signal transducer and activator of transcription 5 (STAT5) then reinforces *Foxp3* expression and supports the survival and expansion of newly generated pTregs [28, 29]. Additional cytokine signals can further reinforce pTreg differentiation and tissue adaptation. IL‐10 promotes human induced Treg differentiation through STAT3‐ and FOXO1‐dependent mechanisms, whereas IL‐33–ST2 signaling supports intestinal Treg accumulation and suppressive function [30, 31]. Conversely, sustained activation of the phosphatidylinositol 3‐kinase (PI3K)‐protein kinase B (AKT)‐mechanistic target of rapamycin (mTOR) pathway and inflammatory IL‐6/IL‐21‐STAT3 signaling oppose stable *Foxp3* induction and favor effector T‐cell differentiation [32, 33, 34, 35]. Recent studies have also identified retinoic acid receptor‐related orphan receptor γt (RORγt)^+^ tolerogenic APCs, including early‐life Thetis cells and PR/SET domain 16 (PRDM16)^+^RORγt^+^ tolerizing dendritic cells (DCs), as specialized populations that present microbiota‐ or food‐derived antigens and activate latent TGF‐β through integrin αvβ8. Disruption of their antigen‐presentation or TGF‐β‐activation programs markedly impairs intestinal pTreg generation and redirects antigen‐specific CD4^+^ T cells toward inflammatory helper‐cell fates [36, 37, 38, 39].

Metabolic cues regulate pTreg differentiation at multiple developmental stages. Even before peripheral antigen encounter, the thymic metabolic environment can shape the capacity of mature naïve CD4^+^ T cells to differentiate into pTregs. Reduced oleic acid availability in thymic epithelial cells enhances disruptor of telomeric silencing 1‐like (DOT1L)‐dependent dimethylation of histone H3 lysine 79 (H3K79) at the ATPase sarcoplasmic/endoplasmic reticulum calcium transporting 2 (*Atp2a2*) locus during thymocyte development. This modification increases the expression of SERCA2 (encoded by *Atp2a2*) and potentiates the calcium‐NFAT1 signaling required for subsequent FOXP3 induction [40]. After antigen encounter in the periphery, locally derived metabolites further shape pTreg differentiation and tissue adaptation. Retinoic acid cooperates with TGF‐β to enhance FOXP3 induction while imprinting the gut‐homing receptors α4β7 and C‐C motif chemokine receptor 9 (CCR9) [41]. Microbiota‐derived short‐chain fatty acids, particularly butyrate, promote pTreg differentiation by increasing histone acetylation at the *Foxp3* locus [42, 43]. Secondary bile acid isoallolithocholic acid directly promotes *Foxp3* induction through NR4A1‐dependent chromatin regulation [44], whereas isodeoxycholic acid acts primarily on DCs to reduce their immunostimulatory activity and thereby favor pTreg generation [45]. Together, these findings support a multistep model in which tolerogenic antigen presentation establishes developmental competence, cytokine and metabolic signals initiate and reinforce FOXP3 expression, and subsequent tissue‐specific adaptation consolidates pTreg stability and tissue‐homing capacity (Figure 1B).

### 2.2 Relative Contributions of nTregs and pTregs

In mice, neuropilin‐1 (NRP1) and Helios are often used to distinguish nTregs from pTregs. High NRP1 and Helios expression is more frequently associated with nTregs, whereas pTregs typically display an NRP1^low/−^ Helios^low^ phenotype [46, 47]. Most Tregs in mice are of thymic origin, although the relative contributions of nTregs and pTregs vary among tissues. Approximately 70%–75% of splenic and peripheral lymph node Tregs express Helios, consistent with a predominance of nTregs in secondary lymphoid organs [3, 4]. By contrast, pTregs are relatively enriched in mucosal tissues, where continuous exposure to dietary antigens, commensal microorganisms, and local tolerogenic signals favors peripheral *Foxp3* induction.

Treg composition also varies with developmental stage and tissue environment. In neonatal and preweaning mice, colonic Tregs are predominantly Helios^+^ nTregs, whereas RORγt^+^ pTregs become markedly enriched after weaning and microbial colonization and constitute a major component of the adult intestinal Treg compartment [22, 48]. Aging further modifies Treg composition by altering thymic output and peripheral Treg maintenance. With age, thymic involution markedly reduces de novo nTreg generation [49]. In parallel, circulating Tregs can re‐enter the thymus, compete for IL‐2, and further suppress the development of newly generated nTregs [50]. Despite this reduction in thymic output, the number of circulating Tregs generally remains stable or increases slightly with age [51]. This relative maintenance of the circulating Treg pool is partly attributable to the ability of both nTregs and pTregs to respond to extrinsic IL‐2 through the high‐affinity IL‐2 receptor complex (IL‐2Rαβγc; CD25/CD122/CD132) and STAT5 signaling, reducing their dependence on continuous thymic production [52, 53]. However, NRP1 and Helios expression can vary with activation state; neither marker alone reliably distinguishes nTregs from pTregs in mice [47, 54].

In humans, no surface or transcriptional marker reliably distinguishes nTregs from pTregs. NRP1 and Helios can be upregulated in pTregs under inflammatory or activated conditions, limiting their value as indicators of cellular origin [55]. Complete demethylation of the *FOXP3* Treg‐specific demethylated region (TSDR) has been used as a more stable indicator of nTreg identity, whereas pTregs typically exhibit partial demethylation [56]. In human peripheral blood, quantitative analysis of FOXP3 TSDR methylation has been developed to identify epigenetically stable nTregs and distinguish them from pTregs or conventional T cells [57]. Tissue‐adapted Tregs have been identified in several human organs, including the gut, adipose tissue, and skin [58]. However, because definitive lineage markers are lacking, the relative contributions of nTregs and pTregs across human tissues remain poorly defined. With aging, the overall frequency of circulating Tregs reportedly remains stable or increases slightly, accompanied by changes in function, metabolism, and epigenetic programming [59]. Thus, aging is associated with changes in Treg abundance and state, but current markers do not permit definitive quantification of age‐related shifts between human nTregs and pTregs.

## 3 Tregs in Health

At steady state, Tregs are distributed in peripheral blood and across lymphoid and non‐lymphoid tissues, forming a dynamic immune‐regulatory network. Circulating Tregs recirculate between the blood and lymph, enter secondary lymphoid organs, and survey local antigen‐presenting environments, restraining inappropriate T and B cell activation and maintaining self‐tolerance without abolishing protective immunity [12, 63]. A fraction of Tregs acquires tissue‐homing and effector programs in lymphoid organs before entering peripheral tissues, where local antigens, cytokines, and metabolites drive additional transcriptional, migratory, and metabolic specialization [7, 64]. Through this compartment‐specific adaptation, tissue Tregs not only limit inappropriate local immune activation but also support barrier integrity, metabolic homeostasis, tissue maintenance, and repair [65, 66]. Thus, physiological Treg function depends on coordinated recirculation, tissue homing, and local adaptation across distinct anatomical compartments, which are discussed separately below.

### 3.1 Lymphoid‐Compartment Tregs: Recirculation, Positioning, and Immune‐Activation Thresholds

Distinct lymphoid compartments possess specialized vascular, stromal, and antigen‐presenting architectures that create distinct environments for Treg trafficking, positioning, and function. Tregs populate the bone marrow, spleen, peripheral lymph nodes, and mucosa‐associated lymphoid tissues, where they localize within hematopoietic stem cell (HSC) niches, T cell zones, B cell follicles, and interfollicular regions [67, 68, 69, 70]. Within these microanatomical sites, Tregs integrate local antigenic, cytokine, chemokine, and stromal cell‐derived signals to calibrate the activation thresholds of neighboring immune cells, while coordinated retention and egress programs maintain dynamic immune homeostasis across lymphoid compartments (Figure 2).

**FIGURE 2:**
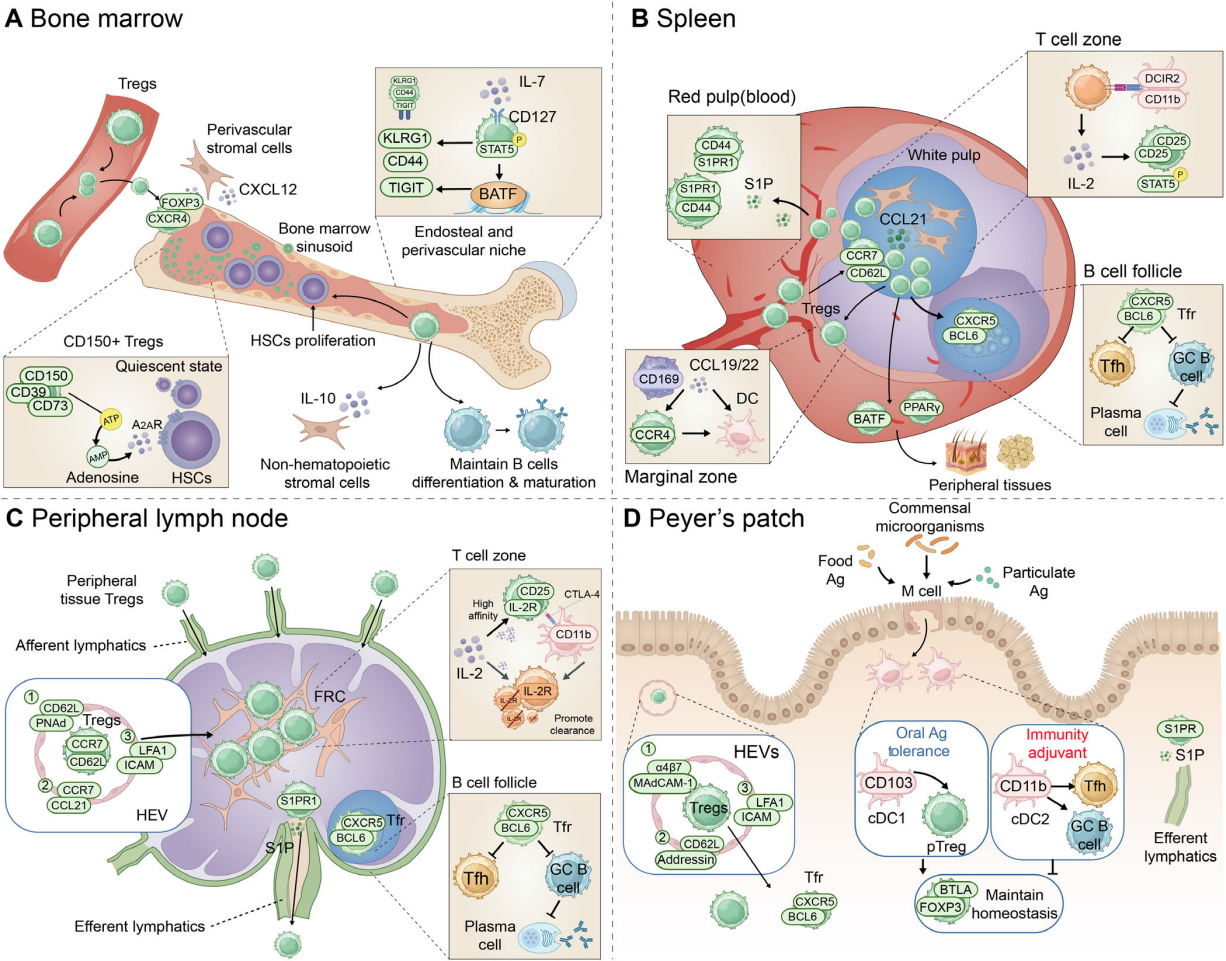
Trafficking, positioning, and homeostatic functions of regulatory T cells in lymphoid compartments. (A) Bone marrow. Circulating Tregs are recruited through the CXCR4–CXCL12 axis and localize near perivascular stromal cells and bone marrow sinusoids. IL‐7–CD127–STAT5 signaling and BATF support the bone marrow Treg phenotype. CD150^+^ Tregs maintain HSC quiescence through CD39/CD73‐dependent adenosine production. Tregs also regulate stromal‐cell function and B‐cell homeostasis. (B) Spleen. Tregs traffic between the white‐pulp, red pulp and marginal zone with CCR7‐dependent positioning and S1P–S1PR1‐mediated recirculation. Local IL‐2 supports Treg maintenance, whereas marginal‐zone Tregs contribute to tolerance to blood‐borne antigens. CXCR5^+^BCL6^+^ Tfr cells restrain follicular and germinal‐center responses. Splenic Tregs can acquire early tissue‐adaptive programs before migration to peripheral tissues. (C) Peripheral lymph node. Circulating Tregs enter through HEVs via CD62L–PNAd, CCR7–CCL21, and LFA‐1–ICAM interactions and localize within T‐cell zones. Local IL‐2 consumption and CTLA‐4‐dependent regulation of APCs restrain self‐reactive T‐cell responses, whereas Tfr cells control follicular responses. Tregs subsequently exit through efferent lymphatics under S1P–S1PR1 control. (D) Peyer's patch. Tregs enter through specialized HEVs and respond to luminal antigens sampled across the follicle‐associated epithelium. Oral antigen exposure favors cDC1‐associated pTreg responses, whereas mucosal adjuvants promote cDC2–Tfh–GC responses. Tfr cells and BTLA‐dependent Treg homeostasis contribute to regulation of local humoral immunity (figure was created with Biorender.com). A2AR, adenosine A2A receptor; Ag, antigen; AMP, adenosine monophosphate; APC, antigen‐presenting cell; ATP, adenosine triphosphate; BATF, basic leucine zipper ATF‐like transcription factor; BCL6, B‐cell lymphoma 6; BTLA, B and T lymphocyte attenuator; CCL, C‐C motif chemokine ligand; CCR, C‐C motif chemokine receptor; CD, cluster of differentiation; cDC, conventional dendritic cell; CTLA‐4, cytotoxic T‐lymphocyte‐associated protein 4; CXCL, C‐X‐C motif chemokine ligand; CXCR, C‐X‐C motif chemokine receptor; DC, dendritic cell; DCIR2, dendritic cell inhibitory receptor 2; FOXP3, forkhead box P3; FRC, fibroblastic reticular cell; GC, germinal center; HEV, high endothelial venule; HSC, hematopoietic stem cell; ICAM, intercellular adhesion molecule; Ig, immunoglobulin; IL, interleukin; IL‐2R, interleukin‐2 receptor; ITIM, immunoreceptor tyrosine‐based inhibitory motif; KLRG1, killer cell lectin‐like receptor G1; LFA‐1, lymphocyte function‐associated antigen 1; M cell, microfold cell; MAdCAM‐1, mucosal addressin cell adhesion molecule 1; P, phosphorylation; PNAd, peripheral node addressin; PPARγ, peroxisome proliferator‐activated receptor gamma; S1P, sphingosine‐1‐phosphate; S1PR, sphingosine‐1‐phosphate receptor; S1PR1, sphingosine‐1‐phosphate receptor 1; STAT5, signal transducer and activator of transcription 5; Tfh, T follicular helper cell; Tfr, follicular regulatory T cell; TIGIT, T‐cell immunoreceptor with Ig and ITIM domains; Treg, regulatory T cell; α4β7, integrin α4β7.

#### 3.1.1 Bone Marrow

Under steady‐state conditions, the bone marrow is the principal site of hematopoietic cell production and differentiation and is also an immune compartment in which Tregs are relatively enriched. In adult specific‐pathogen‐free mice, Tregs account for approximately 40% of bone marrow CD4^+^ T cells, a frequency nearly threefold higher than that observed in the spleen and peripheral lymph nodes [71]. Bone marrow Tregs exhibit a distinct tissue‐adapted phenotype. They are enriched for effector Treg (eTreg)‐associated molecules, including CD44, killer cell lectin‐like receptor G1 (KLRG1), and T cell immunoreceptor with Ig and ITIM domains (TIGIT), and express relatively high levels of the IL‐7 receptor α‐chain CD127. IL‐7 stimulation induces STAT5 phosphorylation in bone marrow Tregs and increases the expression of FOXP3, KLRG1, and other effector‐associated molecules [71, 72]. Treg‐specific deletion of basic leucine zipper transcription factor (BATF) reduces the bone marrow Treg pool and impairs its homeostatic proliferation while attenuating the IL‐7‐induced expression of FOXP3, KLRG1, TIGIT, and CD44 [72].

The recruitment of circulating Tregs to the bone marrow is governed primarily by the C‐X‐C motif chemokine receptor 4 (CXCR4)–C‐X‐C motif chemokine ligand 12 (CXCL12) chemokine axis. Bone marrow Tregs are closely associated with CXCL12^high^ leptin receptor (LEPR)^+^ perivascular stromal cells, and either deletion of CXCL12 in LEPR^+^ stromal cells or Treg‐specific ablation of CXCR4 reduces the number of bone marrow Tregs [68, 73]. Adoptive transfer experiments further showed that bone marrow Tregs preferentially rehome to the bone marrow and can persist locally for at least 8 weeks. Parabiosis experiments demonstrated that approximately 20% of bone marrow Tregs were derived from the partner mouse, indicating that these cells are not strictly sessile tissue residents but are continually replenished from the peripheral circulation [71]. Spatially, intravital imaging studies showed that Tregs accumulate along the endosteal surfaces of the calvarial and trabecular bone marrow and form local clusters around transplanted hematopoietic stem and progenitor cells [74]. Subsequent studies showed that most bone marrow Tregs are positioned near CXCL12^high^LEPR^+^ perivascular stromal cells and bone marrow sinusoids, with CD150^high^ Tregs showing particularly pronounced enrichment around endogenous HSCs [68].

HSC‐proximal CD150^high^ Tregs express high levels of CD39 and CD73, which sequentially convert extracellular ATP and ADP into AMP and adenosine. The resulting adenosine acts on A_2A_ receptors expressed by HSCs, reducing intracellular reactive oxygen species and excessive cell cycle entry, thereby preserving HSC quiescence and steady‐state pool size [68, 75]. Bone marrow Tregs also regulate non‐hematopoietic stromal cells through IL‐10. Mesenchymal stromal cells express relatively high levels of IL‐10Rα within the bone marrow stromal compartment and activate STAT3 in response to IL‐10 produced by bone marrow Tregs. IL‐10 restrains the clonal expansion and partial terminal differentiation of stromal progenitors while increasing the expression of hematopoiesis‐supporting molecules, including LEPR, platelet‐derived growth factor receptor α, stem‐cell factor, and IL‐7. Conversely, IL‐10 neutralization impairs the ability of stromal cells to maintain HSCs and support their long‐term engraftment [71]. Bone marrow Tregs also contribute to the maintenance of the stromal niches required for B cell lymphopoiesis. Treg depletion reduces IL‐7 expression by intercellular adhesion molecule‐1 (ICAM‐1)^+^CD31^−^ perivascular stromal cells and is accompanied by decreases in pro‐B, pre‐B, and mature B cell populations; IL‐7 supplementation substantially restores B cell reconstitution in Treg‐depleted recipients. Similarly, Treg‐specific BATF deficiency reduces the bone marrow Treg compartment and decreases the numbers of B cell progenitors, immature B cells, and mature B cells [72] (Figure 2A).

#### 3.1.2 Spleen

The spleen is an important site where Tregs survey blood‐borne antigens and maintain systemic immune tolerance. CD44^low^CD62L^high^CCR7^high^ central Tregs are predominantly localized within the periarteriolar lymphoid sheaths of the white pulp, which constitute the T‐cell zones, whereas CD44^high^CD62L^low^CCR7^low^ eTregs are relatively enriched in the marginal zone and red pulp. A subset of activated Tregs upregulates B‑cell lymphoma 6 (BCL6) and CXCR5, differentiates into follicular regulatory T (Tfr) cells, and migrates into B‐cell follicles and germinal centers (GCs) [76, 77, 78].

Unlike lymph nodes, the spleen lacks high endothelial venules (HEVs) [70]. Intravital imaging has shown that blood‐borne T cells enter the red pulp and marginal zone before migrating into the white pulp along perivascular stromal tracks through marginal‐zone bridging channels [79]. By responding to stromal‐cell‐derived CCL19 and CCL21, CCR7 guides central Tregs into the white‐pulp T‐cell zones. CCR7‐deficient Tregs can still reach the spleen, but their localization within the white pulp and local STAT5 activation are markedly impaired [76]. The egress of splenic lymphocytes from the white pulp toward the red pulp and bloodstream is regulated by sphingosine‐1‐phosphate (S1P)–sphingosine‑1‑phosphate receptor 1 (S1PR1) signaling [76, 80]. Parabiosis experiments support this recirculation model, showing that CD69^+^S1PR1^−^ Tregs undergo limited exchange between parabionts and exhibit relative long‐term retention, whereas CD69^−^S1PR1^+^ Tregs participate more extensively in recirculation between the spleen and blood [81].

Within the white‐pulp T‐cell zones, dendritic cell inhibitory receptor 2 (DCIR2)^+^CD11b^int^ DCs present self‐antigens via MHC II, stimulating a small population of self‐reactive CD4^+^ T cells to produce IL‐2. Through their high expression of CD25, Tregs capture the limited local supply of IL‐2 and activate STAT5, thereby supporting their survival and homeostatic maintenance [76, 80]. In parallel, Treg CTLA‐4 reduces the surface availability of CD80 and CD86 on APCs via capture and internalization, limiting CD28 costimulation of neighboring conventional T cells [82]. In the marginal zone, CD169^+^ macrophages capture blood‐borne apoptotic cells and produce CCL22, which recruits Tregs and DCs through CCR4. Disruption of CCL22‐CCR4 signaling or reduction of Treg numbers attenuates splenic IL‐10 and TGF‐β responses, enhances the production of inflammatory cytokines, and impairs long‐term tolerance to apoptotic‐cell‐associated self‐antigens [83]. Within B‐cell follicles and GCs, Tfr cells restrain the excessive expansion of T follicular helper (Tfh) cells and GC B cells [77, 78]. In the spleens of healthy mice, B‐cell‐derived self‐antigens can continuously drive spontaneous GC reactions, whereas Tfr cells recognizing the same self‐antigens suppress GC expansion and limit the generation of autoreactive plasma cells and antibodies [84]. Thus, splenic Tregs collectively maintain systemic immune homeostasis by regulating cellular immunity in white‐pulp T‐cell zones, tolerance to blood‐borne self‐antigens in the marginal zone, and humoral immunity within B cell follicles.

The spleen also contains precursors of several non‐lymphoid‐tissue Treg populations. Some splenic Tregs acquire low‐level peroxisome proliferator‐activated receptor γ (PPARγ) expression and early tissue‐migratory features in response to TCR signaling, subsequently migrating to peripheral tissues such as visceral adipose tissue (VAT), skin, and liver, where they undergo further differentiation [6, 85]. The spleen also contains tissue‐Treg precursors whose differentiation toward the non‐lymphoid tissue Treg program is driven by BATF.

#### 3.1.3 Peripheral Lymph Node

At steady state, peripheral lymph nodes are enriched in CD44^low^CD62L^high^CCR7^high^ central Tregs. These cells are predominantly localized within the paracortical T‐cell zone and are distributed along the stromal network formed by fibroblastic reticular cells, where they coexist with conventional CD4^+^ T cells and DCs to establish a local immune‐surveillance microenvironment [76, 86]. Within the paracortical T‐cell zone, a small population of CD4^+^ T cells stimulated by self‐antigens produces IL‐2, promoting the local expansion and suppressive activity of neighboring Tregs. Tregs then use their high‐affinity IL‐2 receptor (IL‐2Rαβγc; CD25/CD122/CD132) to restrict IL‐2 availability to self‐activated T cells and employ CTLA‐4 to attenuate CD80/CD86‐CD28 co‐stimulation, thereby limiting the sustained expansion of self‐reactive T cells and promoting their clearance [76, 87]. Lymph node stromal cells can also express tissue‐derived self‐antigens and present them to naïve CD4^+^ T cells through MHC II molecules, inducing antigen‐specific pTregs in an IL‐2‐dependent manner. These Tregs further restrain Tfh‐cell differentiation and GC B‐cell responses directed against the same self‐antigens [88]. A small subset of Tregs that acquires CXCR5 and BCL6 expression becomes enriched at the T‐B border and within B‐cell follicles, differentiates into Tfr cells, and contributes to the restraint of Tfh‐cell and B‐cell responses [77, 78].

Circulating central Tregs enter peripheral lymph nodes primarily through HEVs. CD62L on Tregs binds to addressins expressed by endothelial cells, mediating initial capture and rolling. Endothelial‐presented CCL21 subsequently activates CCR7, promoting the interaction of lymphocyte function‐associated antigen‐1 (LFA‐1) with ICAM molecules and thereby enabling firm adhesion and transendothelial migration. After entering the lymph node, CCR7 further directs Tregs into the paracortical T‐cell zone. CCR7 deficiency markedly impairs Treg recruitment to lymph nodes, compartmental positioning, and suppressive function in vivo [86, 89]. In addition to entering from the bloodstream, Tregs in peripheral tissues can also migrate through afferent lymphatics into the corresponding draining lymph nodes (DLNs) under steady‐state conditions, thereby conveying information about tissue‐derived antigens and local immune status [90]. Lymph node Tregs primarily enter efferent lymph through the S1P–S1PR1 axis and subsequently return to the bloodstream [91] (Figure 2C). Most Tregs continuously participate in systemic recirculation, and their average residence time within lymph nodes is longer than that of conventional CD4^+^ T cells [92]. In situ photoconversion studies have further shown that approximately 10%–20% of Tregs can persist within the same lymph node for weeks to months while retaining lymph‐node‐specific TCR clonotypes, thereby forming a heterogeneous memory‐like resident population. No single surface marker currently defines this population [67].

#### 3.1.4 Peyer's Patch

Peyer's patches are organized gut‐associated lymphoid tissues located within the wall of the small intestine and comprise the follicle‐associated epithelium and underlying subepithelial dome, interfollicular T‐cell zones, and B‐cell follicles. Unlike peripheral lymph nodes, Peyer's patches lack typical afferent lymphatic vessels and directly survey the intestinal lumen through the follicle‐associated epithelium. M cells provide the principal route for the transepithelial transport of food‐derived, commensal microbial, and particulate antigens. After entering the subepithelial dome, these antigens are captured by DCs and other phagocytes with region‐specific transcriptional and maturation programs and are then conveyed to the interfollicular region to initiate adaptive immune responses [93, 94]. Tregs are distributed both inside and outside GCs in Peyer's patches: extrafollicular Tregs primarily limit local inflammatory responses, whereas intrafollicular Tfr cells participate in regulating GC B‐cell selection and IgA antibody responses [95, 96].

The entry, positioning, and egress of Peyer's patch Tregs generally follow the recirculation mechanisms shared by secondary lymphoid organs, although their homing program displays distinct mucosal features. Circulating lymphocytes enter Peyer's patches through specialized HEVs, where interactions between α4β7 integrin and endothelial mucosal addressin cell adhesion molecule 1 (MAdCAM‐1) cooperate with CD62L and LFA‐1 to mediate cellular capture, rolling, and firm adhesion [97]. After entering the tissue, Tregs are directed toward interfollicular T cell zones or B cell follicles according to their expression of CCR7 or CXCR5, respectively [76, 77]. Lymphocytes subsequently leave through efferent lymphatic vessels under the control of S1P–S1PR1 signaling. Recent studies further indicate that the intestinal microbiota not only helps maintain Peyer's patch cellularity but also regulates the entry of circulating lymphocytes and their S1PR1‐dependent egress [98]. Most Tregs continuously participate in recirculation; however, parabiosis experiments have identified a relatively long‐lived Treg population within Peyer's patches, whose retention is also modulated by the intestinal microbiota [81].

Peyer's patches can also adopt distinct tolerogenic or immunogenic programs according to the immunological context of luminal antigens. Following oral antigen exposure in the absence of an adjuvant, conventional type 1 DCs (cDC1s) and pTregs increase within interfollicular T cell zones. By contrast, the addition of a mucosal adjuvant promotes the accumulation of migratory CD103^+^CD11b^+^ cDC2s, Tfh cells, and IgA responses. These findings indicate that Peyer's patches promote antigen‐specific pTreg responses during non‐inflammatory oral antigen exposure but shift toward a Tfh–GC–IgA‐dominated mucosal immune program in the presence of danger signals [99]. The core function of Peyer's patch Tregs is to coordinate local inflammatory suppression, GC responses, and IgA antibody selection. Depletion of Tregs weakens extrafollicular inflammatory control and disrupts IgA selection within GCs, resulting in alterations in the IgA antibody repertoire and intestinal microbial composition [95]. Recent evidence shows that Treg‐intrinsic B and T lymphocyte attenuator (BTLA) signaling is required to maintain Peyer's patch Treg homeostasis. Treg‐specific deletion of BTLA reduces local Treg numbers and secondarily increases GC B‐cell abundance, indicating that BTLA indirectly restrains GC responses by preserving Treg homeostasis [96] (Figure 2D). Thus, under steady‐state conditions, Peyer's patch Tregs maintain mucosal immune homeostasis by restraining local inflammation, limiting GC B‐cell expansion, and preserving appropriate IgA antibody selection.

### 3.2 Non‐Lymphoid Tissue Tregs: Tissue Adaptation and Local Homeostasis

Tregs populate diverse non‐lymphoid tissues and acquire organ‐adapted phenotypes in response to local antigens, cytokines, metabolites, and stromal‐cell‐derived signals. These tissue‐specific programs enable Tregs to occupy specialized niches, restrain inappropriate local immune activation, and support organ‐specific homeostatic functions, including barrier integrity, metabolic balance, and the maintenance of local cellular niches.

#### 3.2.1 Intestine

The intestine is a major barrier tissue continuously exposed to dietary antigens, commensal microorganisms, and microbial metabolites. To maintain tolerance despite this persistent antigenic pressure, Tregs are highly enriched throughout the intestinal tract, accounting for approximately 20%–30% of CD4^+^ T cells in the lamina propria of the small intestine and colon [65]. The intestinal mucosa provides important sites for the generation, expansion, and functional maturation of pTregs. Compared with lymphoid organ Tregs, intestinal Tregs exhibit more pronounced tissue adaptation and regional heterogeneity.

In the small intestinal lamina propria of adult specific‐pathogen‐free mice, Tregs comprise Helios^+^ nTregs, food antigen‐associated RORγt^−^NRP1^low/−^ pTregs, and a minor fraction of microbiota‐responsive RORγt^+^ pTregs. A subset of nTregs acquires an intestinal tissue‐adapted effector phenotype characterized by GATA‐binding protein 3 (GATA3) and KLRG1 expression [65, 100]. Food‐antigen‐responsive RORγt^−^NRP1^low/−^ pTregs are preferentially reduced by an antigen‐free diet, consistent with their dependence on dietary antigens [22]. Small intestinal Tregs are located predominantly within the lamina propria, with only a minor population residing in the epithelial compartment [101].

The initial induction of food antigen‐specific pTregs occurs primarily in gut‐DLNs. Recent studies have shown that PRDM16^+^RORγt^+^ tolerogenic APCs present food‐derived antigens through MHC II and activate latent TGF‐β through integrin αvβ8. Disruption of the development, antigen‐presenting capacity, or TGF‐β‐activating program of these cells markedly reduces food antigen‐specific pTregs and redirects CD4^+^ T cells of the same antigen specificity toward effector fates, including the Th2 lineage [38, 39]. Within responding T cells, TGF‐β‐SMAD3 signaling cooperates with TCR‐NFAT and IL‐2‐STAT5 signaling to induce and stabilize FOXP3 expression. Retinoic acid further enhances TGF‐β‐dependent pTreg differentiation and induces the gut‐homing receptors α4β7 and CCR9 [41, 66]. Newly generated pTregs subsequently enter the small‐intestinal lamina propria through interactions between α4β7 and MAdCAM‐1 on intestinal vascular endothelial cells, together with CCR9‐mediated responses to epithelial cell‐derived CCL25 [102]. After entering the small intestinal lamina propria, these cells acquire effector and tissue‐adapted programs characterized by the expression of molecules including CD44, inducible T‑cell costimulator (ICOS), CTLA‐4, and IL‐10 [65, 100]. Food antigen‐specific pTregs restrain inappropriate CD4^+^ T‐cell responses directed against the same dietary antigens [101]. Recent evidence further indicates that they limit the excessive homeostatic expansion of food‐reactive CD8αβ^+^ T cells, while permitting a proportion of these cells to acquire protective effector functions during infection with microorganisms carrying cross‐reactive antigens, thereby balancing food tolerance with antimicrobial immunity [39].

In adult specific‐pathogen‐free mice, microbiota‐dependent RORγt^+^ pTregs constitute the major tissue‐adapted Treg subset in the colon, typically accounting for approximately 40%–60% of all colonic Tregs, and an additional 30%–35% display Helios^+^ or NRP1^+^ phenotypes associated with thymic origin [65, 103, 104]. Compared with small intestinal Tregs, colonic Tregs are more dependent on sustained stimulation by microbiota‐derived antigens and on the long‐term maintenance of a local tolerogenic immune environment. Similarly, microbiota‐specific colonic pTregs are induced by PRDM16^+^RORγt^+^ tolerogenic APCs through MHC II‐dependent antigen presentation and integrin αvβ8‐mediated TGF‐β activation [36, 37, 38]. G protein‐coupled receptor 15 (GPR15) is an important homing receptor that directs mouse Tregs to the colonic lamina propria [105]. Following metabolism of dietary L‐tryptophan by host indoleamine 2,3‐dioxygenase 1 (IDO1) and indoleamine 2,3‐dioxygenase 2 (IDO2), the resulting aryl hydrocarbon receptor (AhR) ligands upregulate GPR15 expression in Tregs and consequently promote their accumulation in the colon [106]. After entering the colonic lamina propria, Tregs undergo further maintenance and effector maturation in response to local environmental cues and microbiota‐derived metabolites. In a non‐inflammatory *Helicobacter hepaticus* colonization model, the lamina propria provides a critical microniche in which microbiota‐responsive Tregs acquire a complete effector program, and local IL‐10^+^ eTregs engage in sustained interactions with CD206^+^ macrophages [107]. Short‐chain fatty acids, including butyrate and propionate, promote *Foxp3* induction and colonic Treg homeostasis through histone deacetylase inhibition and free fatty acid receptor 2 signaling [42, 43, 108]. Bile acid metabolites further shape the local Treg‐Th17 balance; most notably, 3‐oxolithocholic acid suppresses Th17 differentiation by directly inhibiting RORγt, whereas other microbiota‐derived bile acids promote Treg generation through the mechanisms described above [45, 109, 110]. Recent studies have additionally identified a stable, terminally differentiated IL‐10^+^ eTreg population that persists long term in the colon, and selective depletion of this population disrupts colonic homeostasis [111] (Figure 3A). Collectively, colonic Treg homeostasis is established through microbiota‐dependent peripheral induction and tissue‐selective homing and is further reinforced by local cellular and metabolic cues within the lamina propria, thereby maintaining immune tolerance to the intestinal microbiota.

**FIGURE 3:**
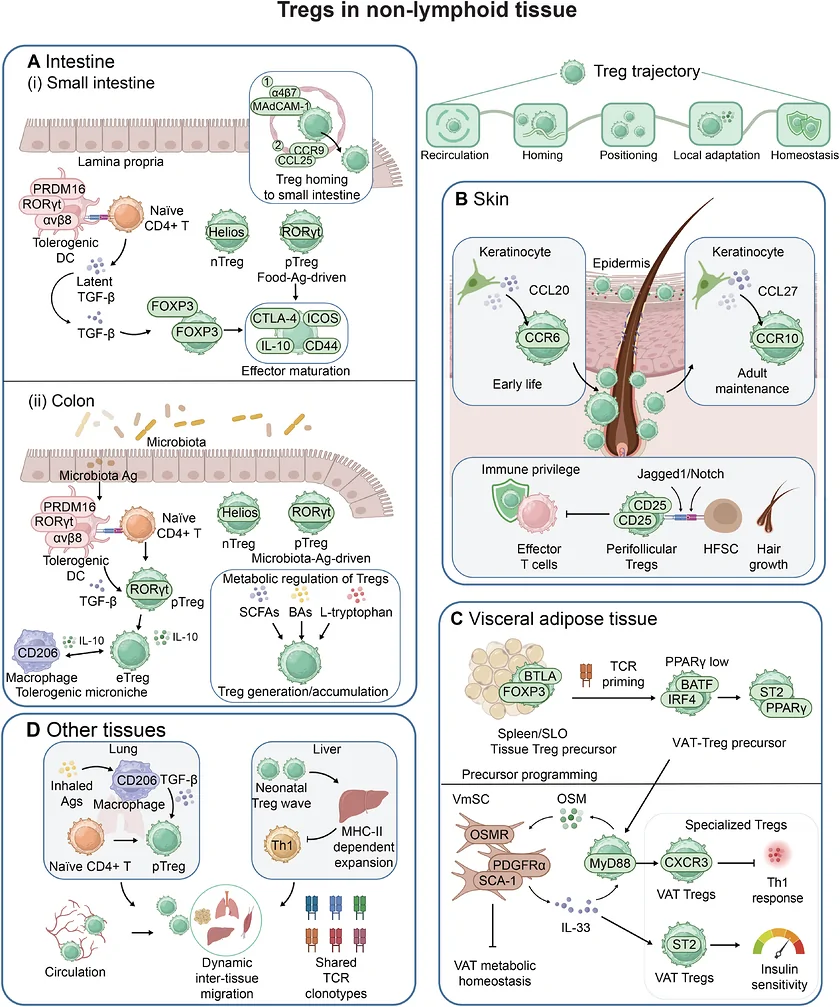
Tissue adaptation and homeostatic functions of regulatory T cells in non‐lymphoid tissues. (A) Intestine. In the small intestine and colon, tolerogenic APCs promote antigen‐specific pTreg generation through TGF‐β‐dependent mechanisms in the MLNs. Intestinal Tregs subsequently acquire tissue‐homing and effector programs, while microbiota‐derived metabolites and local immune‐cell interactions support their accumulation and maintenance. (B) Skin. CCL20–CCR6 signaling promotes early‐life Treg recruitment to perifollicular niches, whereas CCL27–CCR10 contributes to their maintenance in adult skin. Perifollicular Tregs preserve hair‐follicle immune privilege and support hair‐follicle stem‐cell activity. (C) Visceral adipose tissue. TCR‐dependent priming in secondary lymphoid organs initiates a BATF/IRF4–PPARγ‐associated tissue‐Treg precursor program. After entry into VAT, IL‐33–ST2 signaling promotes further differentiation, generating specialized Tregs that restrain Th1 inflammation, support insulin sensitivity, and regulate adipose stromal‐cell differentiation. (D) Other tissues. Tissue‐specific Treg programs also contribute to airway tolerance and neonatal liver homeostasis. Across many non‐intestinal tissues, Tregs undergo dynamic recirculation and inter‐tissue migration, linking tissue homing and local adaptation to organ‐specific immune homeostasis (figure was created with Biorender.com). Ag, antigen; APC, antigen‐presenting cell; BAs, bile acids; BATF, basic leucine zipper transcription factor ATF‐like; BTLA, B and T lymphocyte attenuator; CCL, C‐C motif chemokine ligand; CCR, C‐C motif chemokine receptor; CD, cluster of differentiation; CTLA‐4, cytotoxic T‐lymphocyte‐associated protein 4; CXCR, C‐X‐C motif chemokine receptor; DC, dendritic cell; eTreg, effector regulatory T cell; FOXP3, forkhead box P3; HFSC, hair follicle stem cell; ICOS, inducible T‐cell costimulator; IL, interleukin; IRF4, interferon regulatory factor 4; MAdCAM‐1, mucosal addressin cell adhesion molecule 1; MHC‐II, major histocompatibility complex class II; MLN, mesenteric lymph node; MyD88, myeloid differentiation primary response 88; nTreg, natural regulatory T cell; OSM, oncostatin M; OSMR, oncostatin M receptor; PDGFRα, platelet‐derived growth factor receptor alpha; PPARγ, peroxisome proliferator‐activated receptor gamma; PRDM16, PR/SET domain 16; pTreg, peripheral regulatory T cell; RORγt, retinoic acid receptor‐related orphan receptor gamma t; Sca‐1, stem cell antigen‐1; SCFA, short‐chain fatty acid; SLO, secondary lymphoid organ; ST2, suppression of tumorigenicity 2; TCR, T‐cell receptor; TGF‐β, transforming growth factor‐beta; Th1, T helper 1 cell; Treg, regulatory T cell; VAT, visceral adipose tissue; VmSC, visceral adipose tissue mesenchymal stromal cell; α4β7, integrin alpha 4 beta 7; αvβ8, integrin alpha v beta 8.

#### 3.2.2 Skin

The skin is composed of the epidermis, dermis, and subcutaneous tissue. Under steady‐state conditions, Tregs account for approximately 20% of local CD4^+^ T cells in healthy adult human skin. They are enriched primarily within perivascular and perifollicular niches in the upper dermis and are relatively abundant in hair follicle‐dense regions [112, 113]. A smaller Treg population is also present within the epidermis [114].

The murine skin Treg compartment is established predominantly during early postnatal life. Between postnatal Days 6 and 13, a pronounced wave of Tregs enters the skin. Hair follicle morphogenesis and commensal colonization promote CCL20 production by follicular keratinocytes, thereby recruiting CCR6^+^ Tregs and positioning them around hair follicles [115]. Blocking this neonatal influx impairs the establishment of antigen‐specific tolerance to the same cutaneous commensals in adulthood [116]. Hair follicles therefore constitute a major Treg niche because they simultaneously serve as reservoirs of commensal microorganisms, sources of local chemotactic signals, and sites of epithelial stem‐cell activity.

Tregs in healthy adult human skin are almost uniformly CD45RO^+^ and antigen‐experienced, and most express low or undetectable levels of CCR7. Their limited egress in human skin‐graft models further supports a tissue‐persistent, memory‐like phenotype [113]. Local fate mapping studies in mice similarly demonstrated that a proportion of Tregs present in juvenile skin persists into adulthood, indicating that Tregs entering the skin early in life can form a long‐lived local population [117]. The CCR10‐CCL27 axis contributes to the local accumulation and homeostatic maintenance of skin Tregs; CCR10 deficiency reduces cutaneous Treg abundance and disrupts the balance between local regulatory and effector T cells [118].

Perifollicular positioning enables skin Tregs to maintain both local immune tolerance and epithelial niche homeostasis. In adult mice, selective depletion of skin Tregs is sufficient to induce T‐cell‐mediated inflammation around hair follicles. This protective effect is independent of CTLA‐4 and instead relies primarily on the high‐affinity IL‐2 receptor expressed by Tregs, which competitively limits local IL‐2 availability and thereby protects the hair follicle stem‐cell niche [119]. Skin Tregs also express the NOTCH ligand Jagged1, which promotes hair follicle stem‐cell proliferation and differentiation and supports the transition of hair follicles from the resting to the growth phase [120] (Figure 3B). Thus, under steady‐state conditions, skin Tregs maintain cutaneous homeostasis by promoting commensal tolerance, preserving hair follicle immune privilege, and supporting hair follicle regeneration.

#### 3.2.3 VAT

VAT is both an energy‐storage depot and an endocrine and immunoregulatory organ. Under steady‐state conditions, VAT Tregs form a highly tissue‐adapted eTreg population that contributes to adipose‐tissue and systemic glucose homeostasis by restraining local T‐cell‐mediated inflammation and regulating the differentiation of adipose stromal cells. In the epididymal VAT of adult lean male mice, Tregs progressively accumulate with age and account for approximately 40%–80% of local CD4^+^ T cells, a proportion substantially higher than that observed in lymph nodes, the spleen, or subcutaneous adipose tissue. These cells display a CD44^high^CD62L^low^ effector phenotype and are enriched for tissue‐adaptive molecules, including PPARγ, IL‐33 receptor (ST2), KLRG1, GATA3, and IL‐10. Treg‐specific deletion of PPARγ markedly reduces the VAT Treg compartment and broadly attenuates its lipid‐metabolic and tissue‐effector transcriptional programs, supporting a central role for PPARγ in establishing the mature VAT Treg phenotype and promoting its local accumulation [121, 122, 123].

The tissue‐adaptation program of VAT Tregs is acquired in a stepwise manner. TCR‐transgenic and adoptive‐transfer studies have shown that a subset of pre‐existing pTregs first undergoes TCR‐dependent priming in the spleen, acquiring low‐level PPARγ expression and the capacity to migrate to non‐lymphoid tissues. After entering VAT, these cells respond to local TCR stimulation and IL‐33‐ST2 signaling, completing a mature VAT Treg program characterized by high expression of PPARγ, ST2, and KLRG1. BATF and interferon regulatory factor 4 (IRF4) directly promote the expression of PPARγ and ST2, whereas ST2‐dependent myeloid differentiation primary response 88 (MyD88) signaling supports VAT Treg differentiation, expansion, and maintenance of their transcriptional phenotype [6, 123]. IL‐33 is the cognate ligand for ST2. Under steady‐state conditions, IL‐33 in VAT is produced predominantly by mesenchymal stromal cells, among which the immunocyte‐supporting visceral‐adipose‐tissue mesenchymal stromal cell (VmSC) subsets, VmSC1–3, constitute an important source. Stromal cell‐derived IL‐33 acts on ST2^+^ VAT Tregs to promote their local proliferation and maintain the KLRG1^+^ effector phenotype [124, 125].

The composition of the VAT Treg compartment varies with sex‐associated tissue signals. Male mouse VAT is enriched in the canonical PPARγ^+^GATA3^+^ST2^+^ Tregs. Enhanced CCL2 signaling in male VAT promotes Treg recruitment through CCR2, while androgens favor the development of IL‐33‐producing mesenchymal stromal cells, thereby supporting the expansion of ST2^+^ VAT Tregs. By comparison, CXCR3^+^ Tregs are relatively enriched in female VAT. GATA3, PPARγ, and IL‐33 promote the differentiation of ST2^+^ VAT Tregs, whereas IFN‐γ–T‐box transcription factor (T‐bet) signaling drives the formation of CXCR3^+^ VAT Tregs [126, 127]. These two VAT Treg populations have distinct predominant functions. ST2^+^ VAT Tregs are closely associated with glucose homeostasis; selective disruption of the PPARγ‐dependent ST2^+^ VAT Treg program reduces insulin responsiveness and impairs glucose homeostasis. CXCR3^+^ VAT Tregs predominantly restrain local Th1‐cell‐mediated inflammation. Treg‐specific deletion of T‐bet reduces CXCR3^+^ VAT Tregs and enhances tumor necrosis factor (TNF) and IFN‐γ production by local T cells. Under high‐fat‐diet conditions, this pro‐inflammatory shift further exacerbates glucose intolerance. Thus, CXCR3^+^ VAT Tregs primarily regulate local Th1 cells, whereas ST2^+^ VAT Tregs mainly support adipose‐tissue‐associated glucose metabolic homeostasis [127].

Beyond regulating local immune cells, VAT Tregs can also act directly on adipogenesis. Epididymal VAT Tregs secrete oncostatin M (OSM), which signals through the OSM receptor to inhibit the differentiation of VmSC5 adipocyte precursors into mature adipocytes. Treg‐specific deletion of OSM, or stromal‐cell‐specific deletion of OSM receptor, impairs insulin sensitivity and related metabolic parameters [128] (Figure 3C).

#### 3.2.4 Other Tissues

A well‐defined mechanism of steady‐state immune tolerance operates in the lung. In healthy mice, lung‐resident macrophages express TGF‐β, retinaldehyde dehydrogenase 1 (RALDH1), and RALDH2. Following the uptake and presentation of innocuous inhaled antigens, these macrophages promote the differentiation of antigen‐specific pTregs through the coordinated actions of TGF‐β and retinoic acid (catalyzed by RALDH1/2), thereby attenuating subsequent allergic airway responses to the same antigen [129].

The dura mater of healthy mice contains a small Treg population. Meningeal Tregs limit IFN‐γ production by local lymphocytes and regulate follicular B cell responses. Short‐term depletion of this population induces meningeal inflammation, promotes immune cell entry into the brain parenchyma, and impairs hippocampal neurogenesis [130]. During the first 1–2 postnatal weeks, the mouse liver undergoes a wave of nTreg accumulation. These cells are locally activated and expanded in response to MHC II‐dependent antigenic stimulation, thereby establishing an early postnatal window of Treg‐mediated regulation. Treg depletion between postnatal Days 7 and 11 rapidly induces IFN‐γ‐dominated Th1 inflammation and metabolic disturbance in the liver. Compared with age‐matched splenic Tregs, neonatal liver Tregs exhibit greater proliferative and suppressive capacity, more effectively restrain conventional T‐cell proliferation and IFN‐γ production, and are enriched for T‐bet, PPARγ, and lipid metabolism‐associated transcriptional programs. Through these properties, they support hepatic self‐tolerance and postnatal liver maturation [131]. Muscle Tregs support metabolic homeostasis during exercise by limiting excessive IFN‐γ responses. Small numbers of Tregs are detectable in uninjured skeletal muscle, although their physiological functions have been defined mainly in exercise models. Exercise induces the expansion of muscle Tregs, whereas Treg deficiency increases IFN‐γ production by CD4^+^ T cells, CD8^+^ T cells, and natural killer (NK) cells, directly impairing myofiber mitochondrial respiration and physiological adaptation to exercise [132]. Tregs that maintain tolerance to sperm‐associated antigens are also present in the testes and epididymides of healthy mice. Treg depletion induces infiltration by CD4^+^ T cells and mononuclear phagocytes, promotes the production of antisperm antibodies, and reduces sperm abundance and motility [133].

A recent systematic multi‐organ analysis showed that Treg populations in most non‐intestinal non‐lymphoid tissues are dynamically maintained through transient tissue residency and inter‐tissue migration. Paired single‐cell TCRαβ sequencing revealed extensive clonotype sharing among Tregs derived from the liver, kidney, and pancreas, with many of these clonotypes also detected in peripheral blood and other non‐intestinal tissues. By contrast, intestinal lamina propria Tregs exhibited substantially less TCR clonotype overlap with Tregs from non‐intestinal tissues. These findings are consistent with the possibility that most non‐intestinal tissue Tregs may recognize self‐antigens broadly distributed across multiple organs and may share a cross‐organ TCR repertoire, whereas intestinal Tregs display a more compartmentalized clonal profile [134] (Figure 3D).

## 4 Tregs in Disease: Insufficiency, Maladaptation, and Pathological Suppression

Treg function in disease is highly context dependent. Insufficient recruitment or loss of suppressive stability can aggravate inflammation and tissue injury, whereas persistent Treg activity may instead promote immune suppression in settings such as cancer or chronic infection. These differences are shaped by tissue localization and local environmental signals, and recur across autoimmune disease, transplantation, cardiovascular disease, infection, and cancer.

### 4.1 Recruitment and Positioning of Tregs in Diseased Tissues

Within inflammatory microenvironments, proinflammatory signals such as TNF superfamily member 9 (4‐1BBL), CD40L, glucocorticoid‐induced TNF‐receptor‐related ligand, and lymphotoxin β receptor stimulate vascular endothelial cells to upregulate E‐selectin and P‐selectin, thereby initiating the adhesion cascade that supports Treg recruitment. Tregs expressing P‐selectin glycoprotein ligand‐1 (PSGL‐1) bind endothelial selectins to initiate tethering and rolling [135]. Chemokines such as CXCL10 and CCL5 subsequently activate Treg integrins, enabling firm adhesion to endothelial ICAM‐1 and vascular cell adhesion molecule‐1 (VCAM‐1), followed by transendothelial migration into inflamed tissues. Programmed death‐1 (PD‐1) on eTregs has also been reported to interact with programmed death‐ligand 1 (PD‐L1) on endothelial cells, an effect associated with increased endothelial permeability and enhanced Treg infiltration into inflammatory lesions [136]. After tissue entry, chemokine gradients continue to guide Treg intratissue migration and positioning, with distinct chemokine–receptor axes directing their recruitment and local accumulation in a context‐dependent manner across tumors, injured tissues, transplanted organs, inflammatory lesions, and sites of infection (Table 2). The dominant trafficking pathway is largely determined by the inflammatory milieu and the chemokine profile of the affected tissue. For example, Th1‐skewed inflammation typically induces CXCL9, CXCL10, and CXCL11 expression, thereby recruiting CXCR3^+^ Tregs; Treg recruitment to barrier tissues during type 2 inflammation more commonly involves CCR4‐, CCR8‐, or CCR10‐related pathways; and in Th17‐dominated inflammation, the CCR6‐CCL20 axis can recruit both Tregs and Th17 cells. Thus, pathological outcomes may depend in part on the local numerical and functional balance between Tregs and effector T cells.

**TABLE 2:** Chemokine receptor–ligand axes regulating Treg trafficking in disease.

Receptor	Ligand	Disease context(s)	Target site(s)	Reference
CCR2	CCL2	Breast cancer; HNSCC	Breast tumors and tumor‐draining DLNs; irradiated HNSCC tumors	[137, 138]
CCR4	CCL17	MI	Infarcted myocardium (CCL17 inhibits CCL22‐driven CCR4‐dependent Treg recruitment after MI)	[139]
CCR4	CCL22	T1D; allograft transplantation; breast cancer; ovarian carcinoma	Pancreatic islets; cardiac allografts; islet allografts; breast tumors; ovarian tumors/ascites	[140, 141, 142, 143, 144]
CCR5	CCL5	HCC; pancreatic adenocarcinoma	HCC tumors; pancreatic tumors	[145, 146]
CCR5	CCL3, CCL4, CCL5	Chronic *Leishmania major* infection; pulmonary *Histoplasma capsulatum* infection	Infected dermal lesions; infected lung	[147, 148]
CCR6	CCL20	GN; colorectal cancer; IBD; EAE	Inflamed kidney; colorectal tumors; inflamed colon; inflamed CNS	[149, 150, 151, 152]
CCR7	CCL19	Gastric cancer	Gastric tumors	[153]
CCR7	CCL21	Corneal allograft transplantation	Allograft DLNs	[154]
CCR8	CCL1	AS; MI; breast cancer	Atherosclerotic aortic wall; infarcted myocardium; breast tumors	[155, 156, 157]
CCR9	CCL25	Chronic ileitis	Ileal lamina propria; mesenteric lymph nodes	[158]
CCR10	CCL27	Cutaneous inflammation	Inflamed skin	[159]
CCR10	CCL28	Hypoxic ovarian cancer; HCC; spinal cord injury	Hypoxic ovarian tumors; HCC tumors; injured spinal cord	[160, 161, 162]
CXCR3	CXCL9	Colon carcinoma, thymic lymphoma, and neuroblastoma	Tumors	[163]
CXCR3	CXCL9, CXCL10	GN	Inflamed kidney	[164]
CXCR3	CXCL10	Experimental hepatic inflammation	Inflamed liver	[165]
CXCR6	CXCL16	Cutaneous SCC; biofilm‐associated PJI	SCC tumors; biofilm‐infected joint tissue	[166, 167]

Abbreviations: AS, atherosclerosis; CCL, C‐C motif chemokine ligand; CCR, C‐C motif chemokine receptor; CNS, central nervous system; CXCL, C‐X‐C motif chemokine ligand; CXCR, C‐X‐C motif chemokine receptor; DLN, draining lymph node; EAE, experimental autoimmune encephalomyelitis; GN, glomerulonephritis; HCC, hepatocellular carcinoma; HNSCC, head and neck squamous cell carcinoma; IBD, inflammatory bowel disease; MI, myocardial infarction; PJI, periprosthetic joint infection; SCC, squamous cell carcinoma; T1D, type 1 diabetes; Treg, regulatory T cell.

### 4.2 Adaptation and Pathogenic Reprogramming of Tregs in Disease

After recruitment to lesional tissues, Tregs undergo context‐dependent adaptive remodeling that is further shaped by the local immune microenvironment. Tregs may selectively activate transcriptional programs associated with Th1, Th2, Th17, or Tfh responses, accompanied by upregulation of corresponding chemokine receptors and tissue‐homing molecules, thereby enabling tailored suppression of specific immune responses [168]. In type 1 inflammation, IFN‐γ induces Tregs to express the Th1‐associated transcription factor T‐bet. T‐bet promotes CXCR3 expression, thereby directing Tregs to sites of Th1 inflammation and suppressing Th1 cell proliferation [169, 170]. Notably, the *Il12rb2* promoter in Tregs is typically decorated with the repressive histone mark H3K27me3, which markedly limits their responsiveness to IL‐12‐STAT4 signaling [171]. Thus, Tregs more commonly exhibit a partial acquisition of Th1‐associated programs rather than complete Th1‐lineage differentiation, while retaining FOXP3‐dependent immunosuppressive function. Similarly, in type 2 inflammatory settings and barrier‐tissue microenvironments, Tregs may acquire molecular programs associated with Th2 immunity and tissue adaptation, including IRF4, GATA3, and the IL‐33 receptor ST2, thereby enhancing their residency, reparative activity, and suppressive function in non‐lymphoid tissues such as the skin, intestine, and adipose tissue [172, 173]. In Th17‐type inflammation, Tregs can rely on STAT3‐associated programs to acquire specific control over Th17 responses [174]. During follicular immune responses, Tregs can further differentiate into CXCR5^+^BCL6^+^ Tfr cells, migrate into B‐cell follicles and GCs, and restrain Tfh cell activity, B‐cell responses, and autoantibody production [77].

Under most conditions, Tregs maintain lineage stability, suppressive function, and sustained FOXP3 expression despite diverse inflammatory stimuli [175]. However, under persistent pathological stress conditions such as chronic inflammation, sustained antigenic stimulation, hypoxia, oxidative stress, or metabolic disturbance, Tregs may undergo pathogenic reprogramming and acquire effector‐like transcriptional and functional programs (Figure 4). Depending on the dominant inflammatory milieu, this process can give rise to distinct pathogenic Th‐like Treg states. For example, prolonged IL‐12‐ and IFN‐γ‐dominated signaling can promote the emergence of T‐bet^+^IFN‐γ^+^ Th1‐like Tregs with impaired suppressive activity [176], whereas excessive IL‐4‐STAT6 signaling can drive GATA3^+^IRF4^+^ Th2‐like reprogramming accompanied by production of type 2 cytokines and loss of regulatory function [177]. Sustained IL‐6 and IL‐23 signaling activates STAT3 and promotes the emergence of RORγt^+^IL‐17‐producing Th17‐like Tregs with reduced suppressive capacity and pro‐inflammatory activity [178]. Tregs may also acquire BCL6‐ and CXCR5‐associated follicular‐helper programs and progressively shift toward Tfh‐like states that promote GC responses and humoral inflammation [179]. These processes can be accompanied by reduced FOXP3 expression, increased TSDR methylation, attenuation or loss of suppressive function, and acquisition of a pro‐inflammatory state [12, 180]. With more profound lineage instability, these pathogenic Tregs may even lose FOXP3 expression entirely and become ex‐Tregs with inflammatory or cytotoxic effector properties [181]. Previous studies suggest that nTregs may be more stable than pTregs [182, 183]. For example, in a murine model of corneal allotransplantation, pTregs exhibited functional impairment, whereas nTregs maintained relatively stable function. In high‐risk recipient mice with post‐transplant inflammation, pTregs lost FOXP3 expression and became functionally dysregulated. Adoptive transfer of nTregs after corneal transplantation improved graft survival in high‐risk recipients, whereas adoptive transfer of pTregs failed to confer such therapeutic benefit, suggesting that, in this model, pTregs are more susceptible to reprogramming in inflammatory microenvironments [184].

**FIGURE 4:**
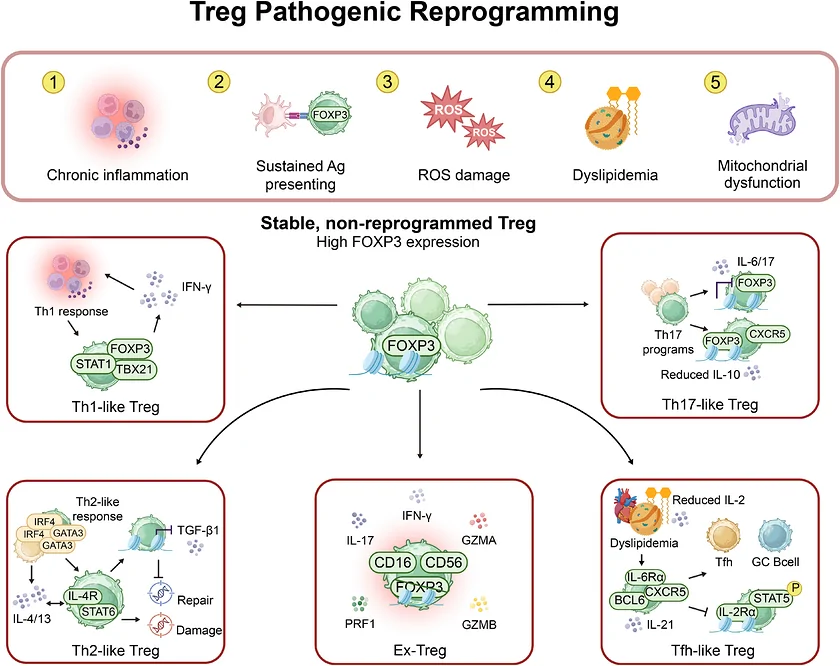
Pathogenic reprogramming and lineage instability of regulatory T cells in disease. Persistent inflammatory and metabolic stress can disrupt the stability of FOXP3^+^ Tregs and promote context‐dependent pathogenic reprogramming. Prolonged type 1 inflammatory signaling drives IFN‐γ‐associated Th1‐like Tregs with impaired suppressive activity, whereas IL‐4–STAT6 signaling in Tregs promotes GATA3‐ and IRF4‐associated Th2‐like reprogramming. Sustained IL‐6/IL‐23–STAT3 signaling favors the emergence of IL‐17‐producing Th17‐like Tregs, while acquisition of BCL6‐ and CXCR5‐associated follicular programs can generate Tfh‐like states that enhance germinal‐center responses. More profound lineage instability can result in loss of FOXP3 expression and generation of ex‐Tregs with inflammatory or cytotoxic features, including IFN‐γ, IL‐17, granzymes, and perforin. Together, these transitions illustrate how persistent inflammatory and metabolic stress can redirect Tregs from stable suppressive states toward dysfunctional or pathogenic phenotypes (figure was created with Biorender.com). Ag, antigen; BCL6, B‐cell lymphoma 6; CD, cluster of differentiation; CXCR5, C‐X‐C motif chemokine receptor 5; FOXP3, forkhead box P3; GATA3, GATA‐binding protein 3; GC, germinal center; GZMA, granzyme A; GZMB, granzyme B; IFN‐γ, interferon gamma; IL, interleukin; IL‐2Rα, interleukin‐2 receptor alpha; IL‐4Rα, interleukin‐4 receptor alpha; IL‐6Rα, interleukin‐6 receptor alpha; IRF4, interferon regulatory factor 4; PRF1, perforin 1; ROS, reactive oxygen species; STAT, signal transducer and activator of transcription; TBX21, T‐box transcription factor 21; Tfh, T follicular helper cell; TGF‐β1, transforming growth factor‐beta 1; Th, T helper cell; Treg, regulatory T cell.

### 4.3 Treg Insufficiency in Autoimmune and Immune‐Mediated Inflammatory Diseases

Previous reviews have summarized the roles of Tregs in disease, but most focus on a single disease or a narrow range of disease subtypes. The present review integrates disorders for which Treg‐targeted interventional trials have been completed or are ongoing, including autoimmune diseases, allograft rejection, CVDs, infectious diseases and malignancies. Mounting evidence indicates that Tregs act as central immune modulators governing disease initiation and progression across these pathologies. Accordingly, this review focuses on disease‐associated phenotypic and functional alterations of Tregs, as well as the molecular mechanisms underlying such changes.

#### 4.3.1 Type 1 Diabetes (T1D)

T1D is a chronic autoimmune disorder predominantly driven by autoreactive T cells, in which the immune system progressively destroys insulin‐producing pancreatic β‐cells and ultimately causes absolute insulin deficiency [185]. Two defects may jointly contribute to the initiation and progression of T1D: insufficient thymic negative selection of pancreatic islet‐reactive T cells, together with deficiency of Tregs. Animal studies strongly support a protective role for Tregs in T1D: specific depletion of Tregs accelerates spontaneous diabetes onset, whereas adoptive transfer or reconstitution of Tregs markedly inhibits disease progression [186, 187]. In human clinical manifestations, monogenic loss‐of‐function mutations in *FOXP3* that abolish Treg homeostasis lead to immune dysregulation, polyendocrinopathy, enteropathy, X‑linked (IPEX) syndrome, which includes early‐onset severe T1D, further supporting the requirement for intact Treg function in maintaining pancreatic β‐cell self‐tolerance [188].

Islet‐autoreactive T cells undergo initial priming within pancreatic DLNs before migrating into pancreatic islets to mediate β‐cell destruction [189]. Accordingly, pancreatic DLNs are an important site of Treg‐mediated protection. Tregs restrain T cell proliferation, activation, and trafficking toward the T‐B cell boundary zone in PLNs [190]. Tang et al. demonstrated that DCs serve as the critical intermediate that transmits Treg‐mediated suppressive signals to T cells in pancreatic DLNs. Stable conjugates between Tregs and T cells are rarely observed; instead, Tregs form direct, sustained contacts with islet antigen‐laden DCs. These long‐lasting Treg‐DC interactions precede DC‐dependent T cell activation [190]. Beyond PLNs, Tregs also execute tolerogenic functions within inflamed pancreatic tissue. CCL22 drives Treg recruitment to the inflamed pancreas and attenuates autoimmune destruction of β cells [140]. NK cells may represent another key cellular target of Treg regulation. In non‑obese diabetic (NOD) mice, conditional Treg depletion accelerates diabetes onset within 3 days, triggering robust NK cell activation and IFN‐γ transcription in insulitic lesions within 7 h. IFN‐γ released by activated NK cells reshapes T cell transcriptomes to drive potent β‐cell cytotoxicity [186].

Current research primarily investigates the changes in the number and function of Tregs in the peripheral blood of patients with T1D. Most studies report normal Treg abundance and function, whereas others report inconsistent increases or decreases [191], and it remains unclear to what extent peripheral blood reflects the functional status of Tregs at the tissue level. A recent study reported an imbalance in specific Treg cell subpopulations among T1D patients [192]. The number of memory Tregs is higher in individuals with T1D and their siblings than in healthy controls. Because increased memory Treg counts are also associated with immune aging, reduced nTreg generation and a shift toward memory phenotypes may complicate the interpretation of this finding [193]. A study analyzing Treg changes in pancreatic DLNs revealed functional defects in Tregs within these lymph nodes in T1D patients [194].

Genetic susceptibility plays a pivotal role in the pathogenesis of T1D, and most susceptibility‐associated single‐nucleotide polymorphisms (SNPs) are located within or near immune‐related genes. Several genes essential for Treg development and suppressive function, particularly IL‐2Rα, IL‐2, protein tyrosine phosphatase non‐receptor type 2, CTLA‐4, and IL‐10, are also significantly associated with T1D [195]. However, only a limited number of studies have directly linked these risk variants to Treg‐intrinsic abnormalities. Through comparative epigenomic profiling of Tregs and conventional T‐cell populations, Ohkura et al. demonstrated that autoimmune disease‐associated SNPs, including those linked to T1D, were preferentially enriched within *FOXP3* TSDR compared with regulatory regions in conventional or effector T cells [196]. In addition to inherited susceptibility, the inflammatory and metabolic environments associated with T1D may secondarily reshape Treg phenotypes and functions. Th1‐associated inflammatory signals can induce Tregs to upregulate T‐bet, CXCR3, and IFN‐γ, thereby promoting the acquisition of a Th1‐like phenotype while retaining measurable suppressive activity [197]. Pancreatic DLNs from patients with T1D also exhibit enhanced Th17 responses accompanied by impaired local Treg‐mediated suppression [194]. Furthermore, insufficient IL‐2 availability within the pancreatic islets of NOD mice reduces CD25 and BCL‐2 expression in Tregs, compromises their survival, and disrupts the balance between Tregs and effector T cells [198]. Recent evidence further indicates that the proportion of TIGIT^+^ Tregs is reduced in patients with T1D, whereas exposure to high glucose concentrations in vitro decreases the frequency of TIGIT^+^ Tregs and, to a lesser extent, CTLA‐4^+^ Tregs [199]. Collectively, both inherited susceptibility and acquired inflammatory and metabolic stress may impair Treg stability and suppressive fitness and thereby contribute to T1D progression.

#### 4.3.2 Systemic Lupus Erythematosus (SLE)

SLE is a prototypic systemic autoimmune disease characterized by loss of immune tolerance to nuclear antigens. The resulting activation of autoreactive T and B cells, autoantibody production, and immune‐complex deposition cause chronic inflammatory injury in multiple organs, including the kidneys, skin, vasculature, and central nervous system [200, 201]. Tregs are key immunoregulatory cells that restrain the sustained amplification of these autoimmune responses and act at multiple stages of SLE pathogenesis. They suppress the activation, expansion, and differentiation of autoreactive CD4^+^ T cells and limit T‐cell help to autoreactive B cells, thereby constraining GC responses, plasma‐cell differentiation, and autoantibody production. Tregs can also suppress local effector T‐cell responses within inflamed tissues and thereby mitigate target‐organ injury [202, 203, 204].

In SLE, Tregs exert their regulatory functions primarily within GCs of secondary lymphoid organs and at inflamed target tissues. Within splenic and lymph‐node GCs, Tfr cells restrain Tfh‐mediated GC B‐cell selection and expansion as well as plasma‐cell differentiation [77, 78]. Recent work further identified a molecular pathway governing this process, showing that IL‐6 can directly act on Tregs to induce CCAAT/enhancer‑binding protein α (C/EBPα) expression; C/EBPα subsequently binds the promoters of *Bcl6* and *Cxcr5* and promotes Tfr differentiation. Treg‐specific deletion of C/EBPα reduces Tfr generation, enhances GC responses, and exacerbates disease manifestations in lupus‐prone mice [205]. In addition to Tfr‐mediated control of Tfh–B‐cell interactions, Tregs can directly restrain autoreactive B cells. In NZB/W F_1_ lupus‐prone mice, Tregs attenuate B‐cell receptor‐induced Ca^2^^+^ signaling through a CTLA‐4‐dependent mechanism, thereby promoting anergy of autoreactive B cells and reducing autoantibody production [3]. Studies in humanized models further support the capacity of Tregs to control lupus‐specific autoimmunity: redirecting SLE patient‐derived Tregs toward the canonical Smith (Sm) autoantigen markedly suppressed antigen‐specific pro‐inflammatory T‐cell responses and halted lupus nephritis progression [206]. The kidney is one of the major target organs affected in SLE, and Tregs also exert important local protective effects within renal tissue. In pristane‐induced lupus, STAT3‐dependent CCR6^+^ Tregs preferentially migrate into renal tissues characterized by Th17‐dominant inflammation and suppress local Th17 responses; Treg‐specific deletion of STAT3 impairs renal Treg recruitment, aggravates lupus nephritis, and increases mortality [207]. Beyond immunosuppression, renal Tregs can directly participate in tissue repair. In murine lupus nephritis, Treg‐specific deletion of amphiregulin (AREG) exacerbates glomerular and tubulointerstitial injury, apoptosis, and fibrosis without substantially altering systemic immune responses, indicating that Treg‐derived AREG can protect renal parenchymal cells through tissue‐reparative mechanisms [208].

Treg abnormalities in patients with SLE are primarily manifested by alterations in subset composition, lineage stability, and suppressive function. In a subset of patients with SLE, particularly those with lupus nephritis, circulating nTregs are expanded and aberrantly express high levels of toll‑like receptor 5 (TLR5) and Fc‑receptor‑like 3. Functional assays further showed that nTregs and memory Tregs from a subset of patients exhibit a reduced capacity to suppress effector T‐cell proliferation, whereas stimulation with the TLR5 ligand flagellin further impairs the suppressive activity of SLE nTregs and can even promote effector T‐cell proliferation, suggesting a potential contribution of disease‐associated microbial signals to Treg functional reprogramming [209]. Tfr cells are also altered in SLE. PD‐1^+^ Tfr cells from patients with SLE display reduced expression of FOXP3, CTLA‐4, and IL‐2Rα together with increased methylation of the *FOXP3* CNS2 region, and consequently show an impaired capacity to suppress Tfh‐cell proliferation [210]. In parallel, circulating Tfh cells are increased, whereas activated Tfr cells are reduced, accompanied by decreased serum IL‐2 levels. In vitro IL‐2 supplementation promotes STAT3 and STAT5 binding to regulatory regions of *FOXP3* and *BCL6*, reduces the repressive H3K27me3 modification at these loci, facilitates the generation of suppressive Tfr cells, and decreases plasmablast formation [211]. IL‐2 deficiency also disrupts Treg homeostasis. CD4^+^ T cells from patients with SLE exhibit impaired IL‐2 production, accompanied by reduced CD25 expression on Tregs and a loss of functionally and metabolically active Treg populations; low‐dose IL‐2 restores CD25 expression and selectively expands Tregs [212]. Beyond cytokine insufficiency, the inflammatory milieu can directly impair Treg function. In active SLE, activated platelets interact with Tregs through the P‐selectin‐PSGL‐1 axis, inducing spleen tyrosine kinase (SYK) phosphorylation and increasing intracellular Ca^2^^+^ signaling, thereby compromising the suppressive functions of both Tregs and Tfr cells [213]. Genetic susceptibility can further impose Treg‐intrinsic defects. Expression of the lupus susceptibility gene pre‑B‑cell leukemia homeobox 1 (PBX1) is reduced in Tregs from patients with SLE, whereas PBX1 deficiency or expression of the lupus‐associated PBX1‐D isoform disrupts rhotekin 2‐mediated cell‐cycle regulation, resulting in reduced Treg abundance, lineage stability, and suppressive capacity while promoting the expansion of inflammatory CD4^+^ T cells [214].

#### 4.3.3 Rheumatoid Arthritis (RA)

RA is a chronic systemic autoimmune disease characterized by persistent synovitis, cartilage damage, and progressive bone erosion. Its immunopathogenesis involves a breakdown of tolerance to self‐antigens, particularly proteins carrying post‐translational modifications such as citrullination, followed by the activation of autoreactive T and B cells and the generation of autoantibodies. As the disease progresses, infiltrating lymphocytes and myeloid cells cooperate with activated synovial fibroblasts to establish a self‐sustaining inflammatory network that ultimately drives chronic synovitis and joint destruction [215]. Tregs are key counter‐regulatory cells in this process, restraining autoreactive lymphocyte responses and limiting the amplification of synovial inflammation and structural joint damage [216]. Experimental studies support this protective role. In collagen‐induced arthritis, induction of collagen II‐specific Tregs in lymph nodes and the spleen markedly attenuated arthritis, synovitis, cartilage degeneration, and bone erosion. Importantly, selective depletion of Tregs substantially diminished this protective effect, whereas adoptive transfer of treatment‐induced Tregs conferred disease protection, supporting the capacity of Tregs to suppress autoimmune arthritis [217].

Tregs exert important regulatory functions within the inflamed rheumatoid joint, where they act on both immune and bone‐remodeling pathways. Tregs accumulate within synovial tissue and can restrain local effector T‐cell responses; experimentally transferred Tregs can be detected rapidly within inflamed synovium, where their accumulation is associated with attenuation of established arthritis [218]. Tregs can also directly inhibit osteoclastogenesis. Tregs suppress macrophage‑colony‑stimulating factor (M‐CSF) and receptor activator of nuclear factor‑κB ligand (RANKL)‐driven osteoclast differentiation and bone resorption predominantly through CTLA‐4‐dependent cell–cell contact, while TGF‐β, IL‐4, and IL‐10 provide additional, but non‐essential, inhibitory signals [219]. However, the inflammatory synovial environment can destabilize this protective program. In autoimmune arthritis, a CD25^low^FOXP3^+^ Treg subset loses FOXP3 expression in response to IL‐6 produced by synovial fibroblasts and differentiates into IL‐17‐producing Th17‐like cells. These cells accumulate within inflamed joints, express high levels of CCR6, CCL20, IL‐23R, and RANKL, and display greater osteoclastogenic activity than conventional Th17 cells, directly linking Treg lineage instability to inflammatory bone destruction [14]. Thus, within the rheumatoid joint, Tregs can restrain local immune activation and osteoclast‐mediated structural damage, but these protective functions depend on the maintenance of Treg identity within the inflammatory synovial milieu.

In patients with RA, Treg abnormalities are characterized by pronounced tissue adaptation and the coexistence of functionally distinct Treg states within inflamed joints. Transcriptomic and epigenomic profiling of synovial‐fluid Tregs has shown that these cells acquire a highly activated eTreg program, characterized by increased expression of the core regulatory molecules *FOXP3*, *CTLA‐4*, and *TIGIT* together with *GITR*, *BATF*, and B‑lymphocyte‑induced maturation protein‑1 (*BLIMP‐1*). This transcriptional adaptation is accompanied by extensive remodeling of H3K27ac‐ and H3K4me1‐marked enhancers and super‐enhancers, indicating extensive epigenetic adaptation of local Tregs within the synovial inflammatory environment [220]. Recent single‐cell analyses of RA synovium identified two tissue‐enriched Treg states with distinct functional properties: highly suppressive CD25^high^CXCR6^+^ Tregs and dysfunctional CD25^low^AREG^+^ Tregs. Synovial macrophage‐derived membrane‐bound TNF supports the metabolic fitness and suppressive activity of CD25^high^CXCR6^+^ Tregs through TNF receptor 2 (TNFR2) signaling, whereas 11β‐hydroxysteroid dehydrogenase type 1 (HSD11B1)‐expressing synovial fibroblasts generate cortisol that promotes the CD25^low^AREG^+^ dysfunctional state; Treg‐derived AREG can in turn reinforce fibroblast inflammatory activation, establishing a maladaptive Treg–stromal feedback circuit [221]. In addition, TNF‐α can directly disrupt the canonical FOXP3 regulatory program. In RA‐derived Tregs, TNF‐α induces protein phosphatase 1 (PP1) expression and activity, resulting in dephosphorylation of FOXP3 at Ser418 within its DNA‐binding domain and consequent impairment of FOXP3 transcriptional activity and Treg suppression; treatment with TNF–α‐specific antibodies reduces PP1 expression, restores FOXP3 phosphorylation, and improves Treg function in patients with RA [222].

#### 4.3.4 Inflammatory Bowel Disease (IBD)

IBD, comprising Crohn's disease (CD) and ulcerative colitis (UC), arises from complex interactions among genetic susceptibility, intestinal dysbiosis, epithelial‐barrier dysfunction, and dysregulated innate and adaptive immune responses to the gut microbiota [223, 224]. Tregs are central to restraining this mucosal immune activation by limiting microbiota‐reactive effector T‐cell responses and maintaining intestinal immune tolerance. Their protective role has been demonstrated in T‐cell–transfer colitis, in which adoptive transfer of Tregs into mice with established colitis induces resolution of the inflammatory infiltrate and restoration of normal intestinal architecture [225]. Genetic studies have also identified a terminally differentiated colonic eTreg population that is indispensable for intestinal homeostasis: selective depletion of these cells induces spontaneous colonic inflammation and increases susceptibility to chemically induced colitis [111].

Tregs exert their regulatory functions at both gut‐DLNs and within the intestinal lamina propria. In transfer colitis, therapeutic Tregs proliferate in the mesenteric lymph nodes and inflamed colon, where they localize in close proximity to CD11c^+^ APCs and pathogenic T cells, allowing them to directly restrain ongoing mucosal immune responses [225]. Their stability within the inflamed intestine depends on local cytokine signals. IL‐10 produced by CD11b^+^ myeloid cells acts directly on Tregs to maintain FOXP3 expression and suppressive activity; accordingly, IL‐10R‐deficient Tregs preferentially lose FOXP3 and fail to control colitis [226]. Intestinal Treg specialization is further reinforced by the transcription factor c‐Maf, which supports the differentiation and function of RORγt^+^ Tregs, promotes Treg‐derived IL‐10, and restrains excessive PI3K‐AKT‐mTORC1 signaling and inflammatory cytokine expression, thereby limiting microbiota‐dependent Th17 and IgA responses [227]. Conversely, inflammatory IL‐23 signaling can directly destabilize colonic Tregs. Treg‐specific IL‐23R signaling reduces their suppressive activity and survival by antagonizing the ATP‐binding cassette subfamily A member 1 (ABCA1)‐mediated cholesterol‐efflux pathway, whereas selective deletion of *Il23r* in Tregs increases their competitive fitness during intestinal inflammation [228]. Recent fate‐mapping studies further revealed a long‐lived, terminally differentiated IL‐10^+^ eTreg population in the colonic lamina propria, most of which express RORγt and acquire reduced dependence on continuous TCR signaling. Acute depletion of this population causes colonic inflammation and exacerbates dextran sulfate sodium (DSS)‐induced disease, although continuous IL‐10 production itself is not required for their protective activity, indicating that these tissue‐adapted Tregs deploy multiple suppressive effector mechanisms [111].

In patients with IBD, Treg abnormalities are particularly evident as inflammatory reprogramming of intestinal Treg subsets and loss of FOXP3 stability rather than as a uniform numerical deficiency. RORγt^+^ Tregs progressively accumulate in the inflamed mucosa of patients with IBD, and a disease‐associated β‐catenin^hi^ subset co‐expresses the inflammatory cytokines IL‐17, IFN‐γ, and TNF. Mechanistically, sustained Wnt–β‐catenin activation is sufficient to reproduce this phenotype: β‐catenin interferes with the cooperative transcriptional activity of *TCF‐1* and *FOXP3* and increases chromatin accessibility at Th17‐ and T‐cell‐activation‐associated loci, thereby epigenetically reprogramming Tregs toward a pro‐inflammatory state [229]. Metabolic alterations in the IBD microenvironment can further destabilize the Treg lineage. Recent studies showed that succinate levels are increased in individuals with IBD, whereas FOXP3 and components of the 2‐oxoglutarate dehydrogenase complex (OGDHc) are reduced in patient Tregs and inversely associated with succinate levels and intestinal inflammation severity. Mechanistically, excess succinate suppresses OGDHc‐dependent production of succinyl‐CoA, reduces FOXP3 succinylation and thereby facilitates FOXP3 ubiquitination and proteasomal degradation, resulting in impaired Treg suppressive function and increased IL‐17 production [230]. Gut dysbiosis provides an additional upstream driver of Treg imbalance: transplantation of microbiota from patients with IBD into germ‐free mice consistently reduces intestinal RORγt^+^ Tregs while expanding Th17 cells and increases susceptibility to experimental colitis [48].

#### 4.3.5 Multiple Sclerosis (MS)

MS is a chronic immune‐mediated disease of the central nervous system (CNS), characterized by inflammatory demyelination, gliosis, axonal injury, and progressive neurodegeneration. Its pathogenesis involves aberrant activation of peripheral autoreactive lymphocytes, particularly Th1‐ and Th17‐polarized CD4^+^ T cells, together with CD8^+^ T cells, B cells, and myeloid cells, which subsequently infiltrate the CNS or act at its barriers to sustain local inflammatory responses against neural and myelin‐associated antigens [231]. Insufficient Treg control can permit sustained expansion and reactivation of encephalitogenic effector T cells, thereby facilitating CNS inflammation and demyelination. Thus, Tregs act as an important checkpoint linking peripheral self‐tolerance with the control of autoimmune inflammation within the CNS.

Tregs exert their protective functions at distinct anatomical stages of CNS autoimmunity. During the peripheral priming phase, DCs promote the generation of myelin‐reactive inducible Tregs in lymphoid tissues by creating an IL‐2‐rich environment, thereby counterbalancing the differentiation and expansion of encephalitogenic effector T cells [232]. Once neuroinflammation is established, myelin‐specific Tregs themselves enter the CNS, although their regulatory efficacy becomes highly dependent on the local inflammatory milieu. In MOG_35–55_‐induced EAE, myelin‐specific Tregs expand in peripheral lymphoid tissues and accumulate within the CNS; however, at disease peak, CNS‐derived encephalitogenic effector T cells that produce IL‐6 and TNF become relatively resistant to Treg‐mediated suppression, allowing autoimmune inflammation to persist despite local Treg accumulation [233]. During the recovery phase, CNS Tregs instead promote disease resolution by restricting local effector T‐cell proliferation, inflammatory cytokine production and motility [234]. Two‐photon imaging of the spinal leptomeninges further revealed that Tregs adopt a confined “repetitive‐scanning” motility pattern around APCs, which limits Th17‐cell access to APCs and consequently reduces their local reactivation. Tregs also directly suppress TCR‐induced Ca^2^^+^ signaling in Th17 cells through a mechanism acting upstream of store‐operated Ca^2^^+^ entry, thereby attenuating Th17 effector activity within the inflamed spinal cord [235]. Beyond immunosuppression, Tregs contribute directly to repair of demyelinated CNS tissue. Treg depletion impairs oligodendrocyte progenitor cell (OPC) differentiation and remyelination, whereas adoptive Treg reconstitution restores these processes; Treg‐derived cellular communication network factor 3 (CCN3) was further identified as a candidate mediator that promotes OPC differentiation and myelination in vitro [236]. This regenerative activity is also affected by aging. Although aged Tregs retain a capacity comparable to that of young Tregs to accumulate within demyelinated CNS lesions, their intrinsic ability to promote OPC differentiation and myelination is reduced. Transcriptomic and functional analyses identified reduced expression of melanoma cell adhesion molecule (*Mcam*) and integrin subunit alpha 2 (*Itga2*) as candidate mechanisms contributing to this age‐associated loss of pro‐remyelinating activity [237].

MS‐associated Treg dysfunction is reflected in the emergence of inflammatory Treg states and impaired maintenance of lineage stability, rather than in a simple reduction in total Treg numbers. In untreated patients with relapsing‐remitting MS (RRMS), the frequency of IFN‐γ‐producing Th1‐like Tregs is increased in peripheral blood, and these cells display an enhanced Th1‐associated transcriptional program together with reduced suppressive activity; exposure of healthy donor Tregs to IL‐12 recapitulates this phenotype by inducing T‐bet and IFN‐γ expression and diminishing suppressive function, whereas withdrawal of IL‐12 or blockade of IFN‐γ partially reverses these changes [238]. Mechanistically, IL‐12 promotes PI3K‐AKT‐dependent phosphorylation and nuclear exclusion of forkhead box O1 (FOXO1), thereby facilitating the acquisition of the Th1‐like Treg phenotype. TIGIT signaling counteracts this process by recruiting SH2 domain‐containing inositol 5‐phosphatase 1, suppressing AKT activation and FOXO1 phosphorylation, and restoring nuclear FOXO1 localization; importantly, TIGIT stimulation reduces T‐bet and IFN‐γ expression and restores the suppressive capacity of Tregs isolated from patients with newly diagnosed RRMS [239]. Recent transcriptomic and epigenomic analyses have identified an additional Treg‐intrinsic mechanism underlying functional instability in MS. A primate‐specific short isoform of PRDM1, PRDM1‐S, is aberrantly upregulated in Tregs from patients with MS and induces SGK1 expression, which destabilizes FOXP3 and compromises Treg suppressive function. This aberrant transcriptional program is accompanied by increased activator protein‑1 (AP‐1)‐ and IRF‐associated chromatin accessibility, suggesting that inflammatory cues may further reinforce Treg dysfunction through epigenetic reprogramming [240].

### 4.4 Allograft Tolerance and Graft Injury

Studies of autoimmunity have established Tregs as important mediators of antigen‐specific immune tolerance, and the same regulatory principle is central to the control of alloimmunity after transplantation. Allogeneic transplantation elicits potent immune responses against non‐self histocompatibility antigens, although the direction of alloreactivity differs fundamentally between solid‐organ transplantation and allogeneic hematopoietic cell transplantation (allo‐HCT). In solid‐organ transplantation, recipient T cells recognize donor alloantigens and differentiate into alloreactive CD4^+^ and CD8^+^ effector cells that mediate T cell‐mediated rejection (TCMR), while Tfh‐dependent activation of donor‐reactive B cells promotes donor‐specific antibody (DSA) formation and antibody‐mediated rejection (ABMR) [241, 242]. Recipient Tregs oppose both arms of this response by restraining alloreactive T‐cell activation and limiting T‐cell help for humoral alloimmunity. Their contribution extends beyond the initial establishment of graft acceptance. In a murine model of kidney‐induced cardiac allograft tolerance, depletion of Tregs 100 days after heart transplantation resulted in rejection of three of four long‐surviving cardiac allografts, indicating that Tregs remain an important component of the maintenance phase of established transplantation tolerance [243]. In allo‐HCT, by contrast, graft‐derived donor T cells recognize recipient HLA and minor histocompatibility antigens. Conditioning‐induced tissue injury and activation of recipient APCs amplify these responses and promote immune‐mediated injury of the gastrointestinal tract, skin, and liver, resulting in graft‐versus‐host disease (GVHD) [244]. Donor‐derived Tregs restrain the activation and expansion of these pathogenic donor T cells; in experimental allo‐HCT, transfer of donor Tregs prevented lethal GVHD while preserving graft‐versus‐tumor activity [245]. Thus, Tregs regulate both major forms of transplantation‐associated alloimmunity, suppressing recipient‐derived anti‐graft responses after solid‐organ transplantation and donor‐derived anti‐host responses after allo‐HCT.

Treg‐mediated regulation is subsequently coordinated across the anatomical sites in which alloimmune responses are initiated and executed. In solid‐organ transplantation, donor‐antigen specificity determines the breadth of Treg‐mediated regulation during alloimmune priming in graft‐DLNs. Experimental studies indicate that Tregs enriched for direct donor allospecificity effectively controlled acute rejection but provided limited protection against chronic allograft injury, whereas additional indirect allospecificity improved control of chronic rejection and transplant vasculopathy [246]. Similarly, introduction of an indirect donor‐reactive TCR into directly alloreactive Tregs enhanced their capacity to protect cardiac allografts, particularly from chronic histopathological injury [247]. Following activation in lymphoid tissues, Tregs accumulate within the allograft, where the local balance between regulatory and effector T cells becomes important for graft protection. In a fully MHC‐mismatched murine cardiac transplantation model treated with anti‐thymocyte globulin and CTLA‐4‐Ig, systemic T‐cell depletion increased the splenic Treg‐to‐effector T‐cell ratio but failed to establish a comparable regulatory environment within the graft. Additional IL‐6 blockade increased intragraft Tregs, reduced CD8^+^ effector‐memory T cells and enhanced IL‐10‐associated regulatory programs, whereas Treg depletion compromised long‐term graft survival [248]. Tregs also regulate humoral alloimmunity through Tfr cells. In murine kidney transplantation, inducible Tfr depletion increased donor‐reactive GC B cells and DSA production and aggravated allograft injury; this response was accompanied by increased IL‐15 production by GC B cells, whereas IL‐15 neutralization attenuated the enhanced humoral response [249]. A comparable dependence on appropriate localization is observed in GVHD. Donor Tregs initially expand in secondary lymphoid tissues and subsequently migrate to recipient target organs. CCR5‐deficient Tregs retain suppressive activity in vitro but show impaired tissue accumulation and reduced protection against GVHD in vivo [250]. After entering target tissues, donor Tregs undergo further local adaptation. Transcriptomic and TCR repertoire analyses demonstrated that these cells acquire organ‐specific transcriptional programs while retaining their regulatory identity, with particularly pronounced adaptation in the colon during acute GVHD [251].

Clinical studies reveal corresponding alterations in Treg abundance, composition, and function, with the clearest evidence in kidney transplantation and allo‐HCT. In kidney transplantation, operationally tolerant recipients who maintain stable graft function after withdrawal of immunosuppression exhibit an enrichment of memory Tregs with greater *FOXP3* TSDR demethylation and preserved suppressive capacity, consistent with expansion of a lineage‐stable regulatory population [252]. Donor specificity further distinguishes Tregs associated with tolerance. Following combined kidney and bone marrow transplantation, pre‐existing donor‐reactive Treg clonotypes expanded during the early post‐transplant period in tolerant recipients but not in a recipient who failed to achieve tolerance [253]. The opposite pattern is observed during humoral rejection. Kidney transplant recipients with DSA‐positive ABMR show a persistent reduction in circulating Tregs together with selective loss of T‐bet^+^CXCR5^+^ Tregs; loss of this population is associated with more pronounced microvascular inflammation and inferior graft survival, while functional assays demonstrate that Tregs suppress Tfh‐driven plasmablast differentiation and antibody production [254]. Analyses of renal allograft biopsies further indicate that lineage identity is more informative than FOXP3 expression alone. Graft‐infiltrating Tregs with stable *FOXP3* TSDR demethylation are associated with less subsequent tissue injury and better graft function [255], whereas increased intragraft *FOXP3* transcript abundance can accompany active rejection and inflammatory cell infiltration without predicting favorable outcome [256]. After allo‐HCT, Treg abnormalities instead predominantly reflect defective immune reconstitution. In a prospective cohort of 215 transplant recipients, circulating Treg frequencies were approximately 40% lower in patients with acute GVHD than in those without GVHD and declined further with increasing disease severity [257]. In chronic GVHD, the underlying defect appears more clearly defined. Thymic generation of nTregs is markedly impaired, leaving a predominantly activated/memory Treg compartment. Although post‐transplant lymphopenia drives vigorous peripheral Treg proliferation, proliferating Tregs are simultaneously more susceptible to Fas‐mediated apoptosis, resulting in a progressive relative Treg deficiency in patients with prolonged CD4^+^ lymphopenia [258]. Limited IL‐2‐dependent homeostatic support further contributes to this imbalance. Low‐dose IL‐2 selectively enhances STAT5 signaling in Tregs, promotes their proliferation and thymic reconstitution, reduces susceptibility to apoptosis and restores the Treg‐to‐conventional CD4^+^ T‐cell balance in patients with chronic GVHD [259]. These observations indicate that transplantation tolerance depends not simply on the number of circulating Tregs, but on the preservation of an alloantigen‐relevant, lineage‐stable, functionally competent Treg compartment capable of maintaining regulatory dominance at the sites of alloimmune activation and tissue injury.

### 4.5 CVDs

Inflammation dynamically shapes the initiation, progression and reparative responses of multiple CVDs, while the inciting stimuli and downstream immune activation differ substantially across disease subtypes [260]. In atherosclerosis (AS), retention and modification of apolipoprotein B (APOB)‐containing lipoproteins within the arterial wall initiate endothelial activation and myeloid‐cell recruitment, while adaptive immune responses against plaque‐associated antigens, particularly APOB, sustain chronic vascular inflammation [261, 262]. Tregs counter these responses by restraining autoreactive T cell and inflammatory myeloid‐cell activity, thereby limiting atherosclerotic lesion development and promoting plaque stability. Consistent with this protective role, depletion of Tregs in *Apoe* ^−^/^−^ and *Ldlr* ^−^/^−^ mouse models increases atherosclerotic lesion burden [263, 264]. Myocardial infarction (MI) elicits a temporally distinct immune response. Cardiomyocyte necrosis induces rapid recruitment of neutrophils and inflammatory monocytes, followed by adaptive immune activation, and timely transition from inflammation to resolution is required for effective scar formation and prevention of adverse ventricular remodeling [265]. Tregs facilitate this transition by suppressing excessive effector T‐cell and monocyte responses, promoting reparative macrophage differentiation and supporting myocardial healing [8]. Across other CVDs, Tregs exert context‐specific protective effects, including suppression of myocardial injury in viral myocarditis, vascular inflammation in abdominal aortic aneurysm (AAA), endothelial dysfunction in hypertension, and inflammation‐driven fibrosis in hypertrophic cardiomyopathy [266, 267, 268, 269]. However, Treg function is not invariably protective during chronic cardiovascular inflammation. In experimental ischemic cardiomyopathy, Tregs that contribute to early post‐MI repair progressively acquire IFN‐γ‐ and TNF‐associated inflammatory features, lose suppressive activity and promote fibrosis and adverse remodeling during chronic heart failure [270]. Similarly, during advanced AS, initially protective APOB‐reactive Tregs can progressively lose their regulatory phenotype and acquire inflammatory effector programs [271]. Thus, Tregs constitute a major counter‐regulatory component of cardiovascular immunity, but their eventual impact depends on disease stage and on the capacity of the local inflammatory and metabolic environment to preserve their regulatory identity.

The context‐dependent activity of Tregs is shaped by their recruitment to diseased cardiovascular tissues and by the local signals that subsequently determine their functional state. Following MI, cardiac antigens are presented in heart‐draining mediastinal lymph nodes, where cardiac‐reactive CD4^+^ T‐cell responses are initiated before Tregs accumulate within the injured myocardium [272]. Within the infarcted myocardium, locally produced chemokines regulate Treg recruitment through distinct receptor axes. CCL3 and CCL4 promote the recruitment of CCR5^+^ Tregs, whereas macrophage‐derived CCL1 recruits CCR8^+^ Tregs to the injured heart [157, 273]. By contrast, CCL17 produced by cardiac macrophages and DCs interferes with CCL22‐driven CCR4‐dependent Treg migration, thereby limiting Treg accumulation within the myocardium [139]. IL‐33 derived from cardiac fibroblasts further supports the expansion and maintenance of cardiac Tregs through ST2 signaling after tissue injury [11]. Within the infarcted myocardium, Tregs restrain pro‐inflammatory monocyte and macrophage responses and promote their transition toward reparative phenotypes. IL‐10 and TGF‐β reduce inflammatory Ly6C^hi^ monocyte/macrophage responses and favor reparative macrophage differentiation [8, 274], while cardiac Tregs additionally contribute to tissue repair through CD39‐CD73‐dependent adenosine production, osteonectin‐mediated scar maturation and paracrine growth‐promoting signals [11, 275, 276]. The importance of local Treg delivery is further supported by fibroblast activation protein (FAP)‐targeted CAR‐Tregs, which enhance Treg accumulation in fibroblast‐rich infarct regions and improve post‐MI repair by suppressing inflammation and pathological fibroblast activation [277]. In viral myocarditis, Tregs engage a distinct protective mechanism in which TGF‐β reduces coxsackievirus and adenovirus receptor expression and thereby limits viral infection and subsequent myocardial injury [267]. In vascular disease, Tregs act predominantly within the inflamed arterial wall. In AS, Treg‐derived IL‐10 and TGF‐β restrain inflammatory T‐cell and macrophage responses, whereas CTLA‐4–dependent suppression of APCs limits local adaptive immune activation [262, 278]. Tregs further modulate lesional macrophage function by enhancing the clearance of apoptotic cells and limiting foam‐cell formation. Treg‐derived IL‐13 promotes macrophage efferocytosis through an IL‐10–Vav guanine nucleotide exchange factor 1 (VAV1)–Rac family small GTPase 1 (RAC1) signaling pathway, whereas Treg‐mediated downregulation of CD36 and SR‐A reduces macrophage uptake of oxidized LDL [264, 278]. In AAA, IL‐10‐producing Tregs reduce macrophage recruitment and local inflammation, while tissue‐adapted aortic Tregs acquire a trefoil factor 1 (TFF1)‐dependent reparative program that contributes directly to vascular repair [279]. In hypertension, Treg‐derived IL‐10 improves endothelial function by suppressing nicotinamide‑adenine‑dinucleotide phosphate (NADPH) oxidase‐dependent oxidative stress [268]. Collectively, these studies indicate that Tregs protect the cardiovascular system through coordinated recruitment to diseased tissues followed by local adaptation, enabling them to combine classical immune suppression with tissue‐specific reparative functions.

Clinical studies indicate that CVD‐associated Treg abnormalities involve changes in abundance, lineage stability and functional phenotype rather than a uniform numerical deficiency. The most detailed evidence is available in coronary artery disease (CAD). Patients with acute coronary syndrome (ACS) exhibit reduced circulating Tregs and lower FOXP3 expression, accompanied by impaired IL‐10 and TGF‐β responses and reciprocal expansion of Th17 immunity [280]. Epigenetic instability may contribute to this regulatory defect. *FOXP3* TSDR methylation increases with CAD severity, whereas exposure of human Tregs to oxidized low‑density lipoprotein (LDL) increases DNA methyltransferase 3A (DNMT3A) and DNMT3B expression, promotes TSDR methylation and reduces *FOXP3* expression, linking the dyslipidemic environment to impaired Treg lineage stability [281]. Disease progression is also associated with qualitative reprogramming of the regulatory repertoire. Human CD16^+^CD56^+^ ex‐Tregs lose suppressive activity and acquire an inflammatory and cytotoxic program characterized by *T‐bet*, *NKG7*, *PRF1*, and *GZMA*, with related transcriptional features observed in CAD [181]. More directly, APOB‐reactive CD4^+^ T cells in patients with mild CAD retain a tissue‐homing effector‐Treg program, whereas this regulatory signature progressively declines with increasing disease severity and is replaced by interferon‐responsive and glycolytic programs in advanced CAD [282]. Treg abnormalities are also evident in myocardial disease. Following acute MI, longitudinal studies have identified changes in circulating Treg abundance together with impaired suppressive activity during post‐infarction follow‐up [283]. In chronic heart failure, both naïve and memory Treg populations are reduced; diminished recent thymic emigrants and T‐cell receptor excision circles indicate impaired thymic output, whereas enhanced spontaneous, IL‐2‐deprivation‐induced and Fas‐mediated apoptosis compromises peripheral Treg maintenance [284]. Patients with dilated cardiomyopathy similarly exhibit reduced Tregs, decreased FOXP3, and glycoprotein‑A repetitions predominant (GARP) expression and diminished TGF‐β1 production, accompanied by impaired suppressive activity of GARP^+^ Tregs [285]. Regulatory defects are also observed in AAA, in which reduced blood FOXP3 expression is associated with more rapid aneurysm expansion [269]. Together, these clinical observations indicate that cardiovascular inflammatory and metabolic stress can impair Treg generation and survival, destabilize FOXP3‐dependent lineage identity and redirect disease‐relevant Treg populations toward inflammatory states.

### 4.6 Infectious Diseases

The pathogenesis of infectious diseases reflects the combined effects of pathogen replication and the ensuing host response. Although innate sensing, antigen presentation, and pathogen‐specific effector responses are indispensable for microbial control, sustained activation of these pathways can disrupt epithelial barriers and cause substantial collateral tissue injury. Tregs play a critical role within this host–pathogen interaction by restraining APCs and innate and adaptive effector populations while coordinating the resolution of inflammation and tissue repair. During acute infection, insufficient accumulation, functional impairment, or inflammatory destabilization of Tregs may therefore aggravate immunopathology. Conversely, during persistent infection, expansion of highly suppressive or pathogen‐specific Tregs can attenuate antimicrobial immunity and facilitate the maintenance of microbial reservoirs. Tregs balance antimicrobial resistance with disease tolerance. They support pathogen control while limiting infection‐induced tissue damage [286, 287].

These opposing effects are determined largely by the anatomical compartment and tissue‐adapted mechanisms engaged by Tregs. During *Streptococcus pneumoniae* infection, pulmonary TNFR2^+^ Tregs selectively restrain IL‐17A‐producing γδ T cells, thereby limiting neutrophilic inflammation, lung injury, and bacterial dissemination into the bloodstream [288]. Tregs also participate directly in the repair of lungs injured by respiratory viruses through the production of AREG. However, this reparative program can itself become dysregulated. In severe viral pneumonia, IL‐6 induces neurogenic locus notch homolog 4 (NOTCH4) expression in lung Tregs, which suppresses IL‐18‐dependent AREG production and compromises tissue repair [289]. A complementary study demonstrated that vimentin sequesters IL‐18 receptor at the distal pole complex of Tregs, restricting IL‐18‐AREG signaling, whereas Treg‐specific vimentin deletion increases Helios^+^IL‐18R^+^ reparative Tregs and accelerates recovery from influenza‐induced lung injury [290]. In the intestinal mucosa, T‐bet^+^ colonic Tregs accumulated in chronically infected *Salmonella* superspreader mice and restrained the expansion of granzyme‐expressing cytotoxic CD4^+^ T cells adjacent to the epithelium. Treg depletion disrupted barrier integrity and reduced host survival without lowering pathogen burden, indicating that these cells maintained disease tolerance while permitting asymptomatic carriage and transmission [291]. By contrast, in healed dermal lesions following *Leishmania major* infection, Tregs recognizing the immunodominant phosphoenolpyruvate carboxykinase epitope exhibited greater suppressive activity than polyclonal Tregs. These pathogen‐specific Tregs produced IL‐10, suppressed local parasite‐specific Th1 responses, and promoted parasite persistence and disease reactivation [292]. Thus, tissue localization and antigen specificity determine whether Tregs primarily prevent collateral injury, maintain barrier function, or establish a locally immunosuppressive niche that favors pathogen persistence.

Recent patient studies further demonstrate that Treg responses are shaped by the infectious agent and disease stage. In chronic hepatitis B virus (HBV) infection, a CTLA‐4^high^ liver‐resident Treg population was preferentially increased during the immune‐tolerant and immune‐active phases of chronic infection. These cells displayed elevated regulatory effector scores and expanded coordinately with PD‐1^+^ exhausted CD8^+^ T cells, with their spatial proximity in the liver confirmed by multiplex immunohistochemistry. This interaction was less evident during acute recovery, suggesting that a compartmentalized suppressive Treg program contributes specifically to chronic viral persistence [293]. A prospective study of 280 hospitalized patients with coronavirus disease 2019 (COVID‐19) similarly found that the frequency of circulating NOTCH4^+^ Tregs increased with disease severity. Patients with both a high proportion of NOTCH4^+^ Tregs and detectable SARS‐CoV‐2 viremia had the greatest disease severity and 90‐day mortality, whereas increased NOTCH4 expression was associated with reduced Treg suppressive activity and diminished AREG production [294]. Treg destabilization has also been demonstrated in SARS‐CoV‐2‐associated multisystem inflammatory syndrome in children, in which enhanced NOTCH1 signaling induced CD22 and disrupted Treg identity through an mTORC1‐dependent pathway, thereby promoting systemic inflammation [295]. Collectively, these findings indicate that an increased Treg frequency may reflect an active suppressive compartment that contributes to pathogen persistence, whereas enrichment of phenotypically dysfunctional or unstable Tregs may coexist with uncontrolled inflammation.

### 4.7 Cancer

Whereas Tregs generally protect tissues from excessive or autoreactive immune responses, their potent suppressive activity can be exploited by tumors to attenuate antitumor immunity and promote tumor progression [296]. Productive antitumor immunity requires the capture and presentation of tumor‐associated antigens and neoantigens by APCs, priming of tumor‐reactive T cells in tumor‐DLNs and subsequent trafficking of effector cells into the tumor. During tumor evolution, this response is progressively constrained by impaired antigen presentation, inhibitory checkpoint signaling and the accumulation of immunosuppressive myeloid and lymphoid populations [296, 297]. Tregs serve as a major cellular mediator of tumor immune suppression and inhibit antitumor immunity at distinct functional stages. They limit DC‐dependent T‐cell priming, restrain the activation and expansion of tumor‐reactive CD4^+^ and CD8^+^ T cells and suppress effector responses within the tumor microenvironment. Consistent with this role, conditional depletion of Tregs can enhance CD8^+^ T‐cell‐ and IFN‐γ‐dependent control of established tumors [298]. Tumor‐infiltrating Tregs are also phenotypically and functionally distinct from their circulating counterparts. Persistent TCR stimulation, together with inflammatory and tissue‐derived signals, promotes their differentiation toward activated eTreg states with enhanced suppressive capacity. Pan‐cancer single‐cell analyses have identified TNFRSF9^+^ activated Tregs as a recurrent population across diverse malignancies, although the relative abundance and transcriptional characteristics of individual Treg states vary substantially among tumor types and patients [299]. Thus, tumors not only recruit Tregs but also promote their local activation and functional specialization, converting a physiological mechanism of immune tolerance into an important barrier to antitumor immunity.

The suppressive activity of tumor‐infiltrating Tregs is reinforced by coordinated recruitment, spatial positioning and local adaptation within the tumor microenvironment. At the tumor‐lymphatic interface, mature regulatory DCs (mregDCs) produce CCL22 and recruit CCR4^+^ Tregs to perilymphatic niches. Treg‐mregDC interactions subsequently restrict DC migration and tumor‐antigen transport to DLNs, thereby limiting antigen availability for the priming of tumor‐reactive T cells [300]. Within the tumor parenchyma, chemokine‐dependent positioning enables Tregs to interact directly with APCs involved in local T‐cell activation. CXCR3^+^ Tregs preferentially localize near CXCL9‐producing BATF3^+^ cDC1s, whereas Treg‐specific loss of CXCR3 reduces these contacts and enhances cDC1‐mediated cross‐presentation and interactions with tumor‐specific CD8^+^ T cells [163]. These spatial interactions also expose Tregs to signals from tumor‐associated myeloid cells that reinforce their effector phenotype. Tumor‐associated macrophage‐derived IL‐23 acts on IL‐23R‐expressing Tregs to sustain FOXP3 expression and BLIMP‐1‐dependent eTreg differentiation, thereby maintaining their suppressive capacity within tumors [301]. Activated intratumoral Tregs subsequently suppress antitumor responses through complementary mechanisms, including CTLA‐4‐mediated regulation of APC costimulation, CD25‐dependent competition for IL‐2, production of inhibitory cytokines, and CD39‐CD73‐dependent generation of extracellular adenosine [296]. The metabolic conditions of the tumor microenvironment further favor Treg persistence and function. In contrast to conventional effector T cells, which are impaired by glucose restriction and lactate accumulation, tumor‐infiltrating Tregs can import lactate through monocarboxylate transporter 1 and use it to support proliferation and suppressive function [302]. Lactate also reinforces the intratumoral Treg program by promoting NFAT1‐dependent PD‐1 expression [303], enhancing TGF‐β‐SMAD3 signaling through moesin lactylation [304] and increasing ubiquitin‐specific peptidase 39 (USP39)‐dependent splicing of CTLA‐4 transcripts [305]. These mechanisms illustrate how chemokine‐guided localization, myeloid‐cell‐derived signals and metabolic adaptation act in concert to establish and maintain highly suppressive Tregs within the tumor microenvironment.

Human studies reveal a recurrent enrichment of activated eTregs within tumors, accompanied by substantial phenotypic heterogeneity across cancer types. Compared with circulating Tregs or Tregs from matched non‐malignant tissues, tumor‐infiltrating Tregs in colorectal cancer (CRC), non‐small‐cell lung cancer (NSCLC), breast cancer, and head and neck squamous cell carcinoma (HNSCC) generally exhibit increased expression of activation and suppressive molecules, including CTLA‐4, ICOS, TNFRSF9, and CCR8 [306, 307, 308, 309]. The relative prominence of these markers, however, varies according to tumor context. TNFRSF9^high^ activated Tregs are particularly prominent in NSCLC and are associated with adverse clinical outcomes [309], whereas CCR8 is strongly enriched in highly suppressive Tregs in human breast tumors [307], and clonally expanded IL1R1^+^ICOS^+^ Tregs with enhanced suppressive activity have been identified in HNSCC relative to site‐matched non‐malignant inflammatory tissues [308]. Despite these phenotypic differences, tumor‐infiltrating Tregs across cancer types converge on activated, tissue‐adapted, and highly suppressive eTreg states. Collectively, these findings indicate that Treg dysregulation in cancer is characterized not simply by increased Treg abundance, but by the selective accumulation and local differentiation of suppressive Treg states shaped by individual tumor microenvironments.

## 5 Treg‐Targeted Therapies: Clinical Progress and Translational Prospects

Over the past decade, clinical translation of Treg‐targeted therapy has developed along four principal strategies: in vivo Treg expansion, adoptive Treg therapy, combination approaches that provide additional immunological or cellular support, and depletion or inhibition of suppressive Tregs in cancer (Table 3). Collectively, clinical experience has shifted the field from simply altering Treg abundance toward improving antigen specificity, tissue localization, lineage stability, and persistence.

**TABLE 3:** Clinical trials of Treg‐directed therapeutic strategies.

Trial ID	Start date	Intervention	Disease/setting	Phase/status	Key reported outcome	Reference
**I. Treg augmentation/restoration**						
**A. Pharmacologic in vivo Treg expansion**						
***A1. Low‐dose IL‐2‐based expansion***						
NCT02932137	May 2016	Low‐dose IL‐2 vs. placebo	SLE	Phase II; Completed	Safe; Tregs expanded; effective in reducing disease severity	[311]
NCT02949492	December 2017	Low‐dose IL‐2	Stable liver transplantation; drug withdrawal	Phase IV; Terminated	Tregs expanded; liver allograft tolerance and immunosuppression withdrawal not achieved	[313]
NCT03312335	August 2018	Low‐dose IL‐2	SLE	Phase II; Completed	Functionally distinct, tissue‐homing Treg subsets expanded	[319]
NCT04077684	September 2019	Low‐dose IL‐2 vs. placebo	Active SLE	Phase IIb; Completed	Safe; Tregs expanded; effective in reducing disease severity	[310]
NCT04077736	November 2019	Low‐dose IL‐2	Relapsing polychondritis	Phase II; Completed	Safe; Tregs expanded; effective in reducing disease severity	[320]
NCT04241601	August 2020	Low‐dose IL‐2 vs. placebo	ACS with residual inflammation	Phase II; Completed	Safe; Tregs expanded; effective in reducing arterial inflammation	[312]
NCT04263831	March 2021	Low‐dose IL‐2	Crohn's disease	Phase I/II; Recruiting	—	—
NCT04065672	October 2021	Low‐dose IL‐2 vs. placebo	Behçet syndrome	Phase II; Completed	Safe; Tregs expanded; effective in reducing disease severity	[321]
NCT05153070	September 2022	Ciclosporin followed by low‐dose IL‐2 vs. followed by placebo	Recently diagnosed T1D with residual insulin secretion	Phase II; Recruiting	—	—
NCT07610538	March 2026	Low‐dose IL‐2 vs. standard care	CAD/MI undergoing CABG	N/A; Recruiting	—	—
***A2. Treg‐selective IL‐2 receptor agonists***						
NCT04119557	November 2019	IL‐2R agonist rezpegaldesleukin vs. placebo	Chronic plaque psoriasis	Phase Ib; Completed	Safe; Tregs expanded; effective in reducing disease severity	[314]
NCT04081350	December 2019	Rezpegaldesleukin vs. placebo	Atopic dermatitis	Phase Ib; Completed	Safe; Tregs expanded; effective in reducing disease severity	[314]
NCT06136741	November 2023	Rezpegaldesleukin vs. placebo	Moderate‐to‐severe atopic dermatitis	Phase IIb; Active, not recruiting	Safe; Tregs expanded; effective in reducing disease severity	[322]
**B. Adoptive Treg cell therapy**						
***B1. Conventional autologous polyclonal Tregs***						
NCT02691247	February 2016	CLBS03/T‐Rex autologous expanded polyclonal Tregs vs. placebo	Recent‐onset T1D	Phase II; Completed	Safe; transient activated‐memory Treg increased; no C‐peptide preservation	[315]
NCT02711826	September 2016	Autologous polyTregs + tacrolimus‐to‐everolimus conversion vs. standard CNI therapy	Kidney transplantation with subclinical allograft inflammation	Phase I/II; Completed	Safe; limited Treg engraftment with some instability; no benefit in graft inflammation	[316]
NCT04691232	February 2021	ER‐TREG01 polyTregs	UC	Phase I; Completed	Safe; Tregs expanded; effective in reducing disease severity	[323]
NCT06708780	April 2023	Autologous polyTregs vs. routine care	T1D	Phase I; Recruiting	—	—
***B2. Allogeneic/UCB‐derived and tissue‐homing Tregs***						
NCT04468971	September 2020	CK0802 UCB‐Tregs	COVID‐19 ARDS	Phase I; Completed	No grade ≥ 3 product‐related toxicity or excess infection; Ineffective	[324]
NCT05423691	December 2022	CK0804 bone‐marrow‐homing UCB‐Tregs + ruxolitinib	Myelofibrosis	Phase I; Recruiting	—	—
NCT05695521	April 2023	CK0803 neurotropic UCB‐Tregs vs. placebo	ALS	Phase I; Active, not recruiting	—	—
NCT06052436	June 2023	Allogeneic nTregs vs. standard care	SIRS/COVID‐19/ARDS	Phase I/II; Recruiting	—	—
***B3. Antigen‐specific or engineered Tregs***						
NCT05234190	January 2022	QEL‐001 autologous HLA‐A2‐specific CAR‐Tregs	HLA‐A2 mismatched liver transplantation	Phase I/II; Active, not recruiting	—	—
NCT06201416	March 2024	SBT777101 CAR‐Tregs	RA	Phase I; Recruiting	—	—
NCT06566261	September 2024	ABA‐101 TCR‐engineered Treg	Progressive multiple sclerosis	Phase I; Active, not recruiting	—	—
NCT06361836	October 2024	SBT777101 CAR‐Tregs	Hidradenitis suppurativa	Phase I; Active, not recruiting	—	—
NCT06919354	September 2025	GNTI‐122 engineered autologous Tregs	Recently diagnosed T1D	Phase I; Recruiting	—	—
NCT07473154	April 2026	QEL‐005 autologous CD19 CAR‐Tregs	Systemic sclerosis/RA	Phase I/II; Recruiting	—	—
***B4. Hybrid Treg/Th2‐like cell products***						
NCT04482699	December 2020	Allogeneic hybrid Treg/Th2 T cells	Severe COVID‐19/ARDS	Phase I/II; Terminated	The eligible patient population changed	—
NCT04220190	January 2025	RAPA‐501 autologous hybrid Treg/Th2‐like T‐stem cells	ALS	Phase II/III; Recruiting	—	—
**C. Combination and transplantation/graft‐engineering platforms**						
***C1. Treg plus cytokine or immunomodulatory therapy***						
NCT03241784	May 2016	Repeated autologous polyTregs + low‐dose IL‐2	ALS	Phase I; Completed	Safe; Treg numbers/function increased; ALS progression slowed or stabilized	[325]
NCT02772679	August 2016	Autologous polyTregs + low‐dose IL‐2	T1D	Phase I; Completed	Well tolerated; transferred/endogenous Tregs expanded, but NK, MAIT and CD8^+^ T cells were activated; no C‐peptide preservation	[326]
NCT06688331	March 2025	Tregs + rituximab vs. Tregs + placebo vs. rituximab + Treg sham vs. dual placebo/sham	Pre‐diabetic state/T1D prevention	Phase II; Recruiting	—	—
NCT06920199	June 2025	Donor Tregs + low‐dose IL‐2	Refractory cGVHD	Early phase I; Recruiting	—	—
***C2. Treg‐enabled chimerism/HSPC platforms***						
NCT03867617	August 2019	Recipient polyTregs + donor marrow + tocilizumab vs. standard transplant care	HLA‐mismatched living‐donor kidney transplantation	Phase I/II; Active, not recruiting	Safe; Treg‐functional, chimerism achieved, immunosuppression reduced	[327]
NCT03943238	February 2020	Donor CD34^+^ HSPCs + recipient Tregs	Kidney transplantation	Phase I; Recruiting	—	—
NCT05088356	September 2021	Donor Tregs + CD34^+^ HSPCs + Tcons	GVHD prevention	Phase I; Active, not recruiting	—	—
***C3. Treg‐enriched engineered graft platforms (Orca)***						
NCT03802695	April 2019	Orca‐Q graft across six donor/conditioning cohorts	GVHD prevention	Phase I; Recruiting	—	—
NCT04013685	November 2019	Orca‐T CD34^+^ HSPC + Tregs + Tcons + tacrolimus	Advanced hematologic malignancies; allogeneic HCT	Phase I; Active, not recruiting	—	—
NCT05316701	June 2022	Orca‐T + tacrolimus vs. unmanipulated graft + tacrolimus/methotrexate	GVHD prevention in hematologic malignancies	Phase III; Active, not recruiting	Safe; early Treg elevation post‐transplant with superior GVHD control	[317]
NCT05507827	August 2022	Orca‐T graft + donor CD19/CD22 CAR T cells	B‐ALL after HSCT	Phase I; Active, not recruiting	—	—
NCT06195891	May 2024	TMLI/fludarabine/melphalan conditioning + Orca‐T ± thiotepa/tacrolimus	AML/ALL/MDS allogeneic HCT	Phase I; Recruiting	—	—
NCT06551584	December 2025	Orca‐T + tacrolimus + ruxolitinib	Advanced hematologic malignancies; HLA‐mismatched allogeneic HCT	Phase I; Recruiting	—	—
**II. Treg depletion/inhibition in cancer**						
**D. CCR8‐directed intratumoral Treg targeting**						
***D1. Anti‐CCR8 monoclonal antibody‐based strategies***						
NCT04895709	May 2021	Imzokitug, anti‐CCR8 mAb vs. + nivolumab vs. + docetaxel vs. + pumitamig	Advanced solid tumors	Phase I/II; Recruiting	—	—
NCT05007782	August 2021	Denikitug, afucosylated anti‐CCR8 mAb vs. denikitug + zimberelimab	Advanced solid tumors	Phase I; Active, not recruiting	—	—
NCT05537740	October 2022	Anti‐CCR8 mAb alone vs. anti‐CCR8 mAb + pembrolizumab	Advanced solid tumors	Phase I; Active, not recruiting	—	—
NCT05635643	December 2022	Tagmokitug, afucosylated anti‐CCR8 mAb vs. tagmokitug + toripalimab	Advanced solid tumors	Phase I; Active, not recruiting	—	—
NCT06657144	April 2025	Tagmokitug + toripalimab	Advanced or metastatic solid tumors	Phase I; Recruiting	—	—
NCT07362186	April 2026	Anti‐CCR8 mAb + toripalimab vs. paclitaxel	CCR8+ gastric/GEJ adenocarcinoma	Phase III; Recruiting	—	—
***D2. CCR8‐targeted antibody‐drug conjugate***						
NCT07205718	November 2025	Anti‐CCR8 antibody–drug conjugate	Advanced/metastatic solid tumors with CCR8+ Tregs	Phase I/II; Recruiting	—	—

Abbreviations: ACS, acute coronary syndrome; ALL, acute lymphoblastic leukemia; ALS, amyotrophic lateral sclerosis; AML, acute myeloid leukemia; ARDS, acute respiratory distress syndrome; B‐ALL, B‐cell acute lymphoblastic leukemia; CABG, coronary artery bypass grafting; CAD, coronary artery disease; CAR, chimeric antigen receptor; CCR8, C‐C motif chemokine receptor 8; CD19, cluster of differentiation 19; CD22, cluster of differentiation 22; CD34, cluster of differentiation 34; CD8, cluster of differentiation 8; cGVHD, chronic graft‐versus‐host disease; CNI, calcineurin inhibitor; COVID‐19, coronavirus disease 2019; GEJ, gastroesophageal junction; GVHD, graft‐versus‐host disease; HCT, hematopoietic cell transplantation; HLA, human leukocyte antigen; HSCT, hematopoietic stem cell transplantation; HSPC, hematopoietic stem and progenitor cell; IL‐2, interleukin‐2; IL‐2R, interleukin‐2 receptor; mAb, monoclonal antibody; MAIT, mucosal‐associated invariant T cell; MDS, myelodysplastic syndrome; MI, myocardial infarction; N/A, not applicable; NCT, ClinicalTrials.gov identifier; NK, natural killer; nTreg, natural regulatory T cell; polyTreg, polyclonal regulatory T cell; RA, rheumatoid arthritis; SIRS, systemic inflammatory response syndrome; SLE, systemic lupus erythematosus; T1D, type 1 diabetes; Tcon, conventional T cell; TCR, T‐cell receptor; Th2, type 2 helper T cell; TMLI, total marrow and lymphoid irradiation; Treg, regulatory T cell; UC, ulcerative colitis; UCB, umbilical cord blood.

In vivo Treg expansion is readily scalable and avoids individualized cell manufacturing. Low‐dose IL‐2 has provided the clearest proof of principle, expanding Tregs and producing clinical or anti‐inflammatory signals in SLE, Behçet syndrome, and CVDs [310, 311, 312]. However, in the LITE liver‐transplant trial, expansion of circulating Tregs failed to establish sufficient donor‐specific regulation within the graft, and immunosuppression withdrawal was unsuccessful [313]. Treg‐biased IL‐2 agonists therefore seek greater cellular selectivity. Rezpegaldesleukin, a Treg‐selective pegylated IL‐2 receptor agonist, induced sustained Treg expansion and early clinical responses in atopic dermatitis and psoriasis [314], although responses across diseases remain variable. These findings indicate that increasing circulating Treg numbers alone may be insufficient when antigen specificity, tissue access, or functional fitness remains limiting.

Adoptive therapy provides greater control over Treg phenotype and dose, but experience with polyclonal products has similarly revealed limitations. In recent‐onset T1D, the randomized T‐Rex trial showed that expanded polyclonal Tregs were safe but did not preserve β‐cell function, with cell‐product quality appearing more informative than expansion magnitude [315]. Likewise, polyclonal Tregs did not significantly improve subclinical kidney‐allograft inflammation, highlighting the importance of durable engraftment and functional stability after transfer [316]. Clinical development is therefore expanding beyond conventional polyclonal products toward tissue‐derived or allogeneic Tregs and antigen‐directed engineered platforms. Current approaches include umbilical cord blood‐derived and thymus‐derived Tregs, TCR‐engineered Tregs and HLA‐A2‐specific CAR‐Tregs (Table 3). Compared with polyclonal products, these approaches aim to concentrate regulatory activity at disease‐relevant sites rather than broadly suppress immunity. However, most of these approaches remain at an early stage of clinical development, and their therapeutic efficacy has yet to be established.

Combination Treg‐based therapies aim to reinforce regulatory activity by providing a more favorable environment for transferred Tregs or by incorporating them into defined cellular grafts. The most important advance is Tregzi (Orca‐T), which became the first FDA‐approved Treg‐based immunotherapy in 2026 for matched‐donor myeloablative hematopoietic stem cell transplantation (HSCT) in adults with hematological malignancies. Unlike stand‐alone Treg infusion, Orca‐T integrates purified donor Tregs with hematopoietic progenitors and a controlled conventional T‐cell component. In the phase III Precision‐T trial, this defined cellular architecture substantially improved survival free from moderate‐to‐severe chronic GVHD compared with conventional transplantation [317]. Its success therefore supports precise control of graft cellular composition, rather than nonspecific Treg administration alone, as an important principle of Treg‐based therapy.

In cancer, the therapeutic objective is reversed: suppressive intratumoral Tregs must be selectively disrupted while systemic immune tolerance is preserved. Broad targeting has exposed this challenge. For example, the anti‐CCR4 antibody mogamulizumab can deplete CCR4^+^ eTregs, but its limited cellular selectivity may also affect CCR4‐expressing central‐memory CD8^+^ T cells and thereby compromise antitumor immunity [318]. Current programs therefore increasingly target tumor‐enriched CCR8^+^ Tregs, including imzokitug (NCT04895709), denikitug (NCT05007782), tagmokitug (NCT05635643 and NCT06657144), and other anti‐CCR8 antibody‐based strategies (NCT05537740, NCT07205718, and NCT07362186). These agents are being developed with greater tumor selectivity through optimized depletion mechanisms, antibody–drug conjugation, and selected combinations with PD‐1 blockade. Denikitug, for example, is an afucosylated anti‐CCR8 monoclonal antibody being evaluated alone and with anti‐PD‐1 therapy in advanced solid tumors. Whether selective CCR8^+^ Treg depletion translates into durable clinical benefit remains to be established.

## 6 Conclusions and Future Perspectives

Over the past two decades, accumulating evidence has established that Tregs function across multiple levels, ranging from systemic immune tolerance to tissue homeostasis and injury repair [3]. Thymic and peripheral developmental pathways jointly generate a Treg repertoire with distinct antigen specificities, enabling responses to self, environmental and tissue‐associated antigens. After entering non‐lymphoid tissues, Tregs further integrate TCR stimulation with inflammatory, metabolic and extracellular matrix‐derived signals to acquire functional states adapted to local requirements [12, 15]. This adaptability can have divergent consequences across disease settings. Autoimmune and chronic inflammatory environments may impair Treg suppressive function and, in some contexts, compromise FOXP3 stability or induce effector T‐cell‐like features; whether all such changes represent bona fide lineage conversion remains debated [12, 175, 180, 181, 183]. By contrast, the tumor microenvironment can reinforce the stability and suppressive activity of eTregs through myeloid cell‐derived signals, metabolic reprogramming and spatial organization, thereby constraining antitumor immunity [300, 302, 304, 305, 328, 329, 330]. Treg function should therefore be interpreted in relation to antigen specificity, tissue localization, functional state and disease stage.

Accurately defining the identity and fate of human Tregs remains a major challenge. FOXP3 and CD25 can be transiently expressed by activated conventional CD4^+^ T cells, whereas definitive markers that distinguish human nTregs from pTregs remain unavailable [12, 15]. Cross‐tissue single‐cell studies have further revealed pronounced tissue‐specific and clonal compartmentalization of immune cells, indicating that peripheral blood analysis cannot fully capture the states of Tregs within diseased tissues [331]. Future studies should incorporate longitudinal, paired sampling of peripheral blood, lymph nodes and diseased tissues. Integrating single‐cell transcriptomics, chromatin accessibility profiling, TCR clonotype tracking, demethylation analysis of TSDR and spatial omics should help distinguish transient environmental adaptation from stable tissue differentiation and bona fide lineage instability [332].

Clinical trials indicate that increasing Treg abundance does not consistently translate into clinical benefit. The ONE Study provided initial evidence supporting the safety and feasibility of Treg‐based cell therapy in kidney transplantation [333]. However, adoptive transfer of expanded polyclonal Tregs failed to provide sustained preservation of pancreatic islet function in a randomized phase II trial involving children with recent‐onset type 1 diabetes [315]. Although IL‐2 and Treg‐selective IL‐2 receptor agonists can expand circulating Tregs, their therapeutic effects vary considerably across diseases [312, 314, 334, 335]. Next generation therapies should prioritize antigen specificity, FOXP3 stability, tissue homing and in vivo persistence. Therapeutic efficacy should be evaluated using TCR clonotype persistence, TSDR demethylation, tissue‐level pharmacodynamic responses and organ‐specific functional outcomes [16, 17, 336, 337]. In cancer, therapeutic strategies should selectively restrain activated intratumoral Tregs while preserving peripheral immune tolerance as far as possible [328]. The choice of Treg subset, target tissue and timing of intervention should therefore be tailored to the disease stage and underlying tissue context. Overall, Tregs integrate developmental origin, antigenic stimulation and tissue‐derived signals to exert context‐dependent effects on immune tolerance, tissue homeostasis and disease progression. Defining this regulatory network more precisely will provide a foundation for selectively enhancing or restricting Treg function across different diseases.

## Author Contributions

M.L. and S.Z. drafted the manuscript. M.L. and T.T. generated the figures and tables. T.W. and X.C. designed the work and revised the manuscript. All authors have read and approved the final manuscript.

## Funding

This work was supported by the Noncommunicable Chronic Diseases—National Science and Technology Major Project (No. 2025ZD0548400, 2023ZD0504600 and 2023ZD0504604), the Natural Science Fund of Hubei (No. 2023AFA113).

## Ethics Statement

The authors have nothing to report.

## Conflicts of Interest

The authors declare no conflicts of interest.

## Data Availability

The authors have nothing to report.
